# Iron uptake by *Escherichia coli* in urinary tract infections and urosepsis

**DOI:** 10.1371/journal.pone.0326251

**Published:** 2025-06-26

**Authors:** Beata Krawczyk, Paweł Wityk, Agnieszka Laskowska, Anna Stanisławska-Sachadyn, Joanna Raczak-Gutknecht, Małgorzata Waszczuk-Jankowska, Magdalena Burzyńska, Marek Bronk, Tomasz Majchrzak, Michał J. Markuszewski

**Affiliations:** 1 Department of Biotechnology and Microbiology, Faculty of Chemistry, Gdańsk University of Technology, Gdańsk, Poland; 2 Department of Biopharmaceutics and Pharmacodynamics, Medical University of Gdańsk, Gdańsk, Poland; 3 Clinical Microbiology Laboratory, Medical University of Gdańsk, Gdańsk, Poland; 4 Department of Analytical Chemistry, Faculty of Chemistry, Gdańsk University of Technology, Gdańsk, Poland; Texas A&M University, UNITED STATES OF AMERICA

## Abstract

The pathogenesis of urosepsis in uncomplicated and community-acquired urinary tract infections (UTIs) caused by *Escherichia coli* was studied. We hypothesized that siderophores involved in iron uptake may determine bacterial adaptation to the urine and blood environment in patients with UTI, leading to urosepsis. *E. coli* isolates were compared from urosepsis patients (cases) and UTI (control group). The patterns of bacterial DNA fingerprints isolated from the blood and urine of patients with urosepsis were compared by PCR melting profile method to detect urosepsis and exclude nosocomial infection. Chromium Azurol S (CAS) assay and RT-qPCR were used to investigate the expression levels of siderophore genes in artificial urine and M9 supplemented with whole blood. *E. coli* isolates from artificial urine were proteomically analysed. The pro-inflammatory factors IL-2,4,6,8,10, IFN-gamma, TNF-alpha and CRP were quantified in the patients’ serum. PCR detected and co-occurrence of enterobactin, aerobactin and yersiniabactin was significantly more frequent in the urosepsis (P = 0.0039) and with the *iha* gene may represent markers of urosepsis risk. Aerobactin was dominant in urosepsis (P = 0.03), but its expression in artificial urine was twice higher than in blood (P = 0.03). When cultured in artificial urine, the expression of *entC* was significantly higher (P = 0.029), while the expression of *iro-2*, *iucA* and *iroB* were lower in strains obtained from urosepsis (P = 0.007; P = 0.030; P = 0.012; respectively) as compared to the UTI group. Ferritin-1, iron uptake system component EfeO, ferrous iron transport protein B, nitrate/nitrite response regulator protein NarL, protein HemY, ferrienterobactin receptor FepA, lipopolysaccharide export system protein LptA and 2Fe-2S ferredoxin were found by proteomic analyses in urosepsis only. A positive association of IL-6,8,10, TNF and CRP proteins with urosepsis was observed. In conclusion, risk factors for UTI-related sepsis may be related to the iron uptake system, and genetic and proteomic profiles may help identify them.

## Introduction

A urinary tract infection (UTI) is defined as bacteriuria with ≥10^5^ bacterial CFU/ml of midstream urine in combination with clinical symptoms [1]. The most common pathogen isolated from UTIs is *Escherichia coli.* If not properly treated in time, UTIs can lead to a bloodstream infection or even sepsis [2,3]. A bloodstream infection caused by bacteria from the urinary tract is called urosepsis [4]. 9–31% of cases of urosepsis have a severe outcome, although the cause is not fully understood.

*E. coli* shows significant genomic plasticity, with a well-conserved core genome and highly variable and heterogeneous accessory genetic elements due to multiple horizontal gene transfer (HGT) events [5,6]. Virulence factors (Vfs) encoding genes of uropathogenic *E. coli* (UPEC) belong to the group of genes that cover a wide range of functions, from increased motility and adhesion to toxin secretion, immune evasion and nutrient acquisition [7–9]. Bacteria also form characteristic small-molecule metal chelators, namely siderophores, that have a higher affinity for metal ions than many host proteins. Strains of UPEC can synthesize 4 types of siderophores, namely enterobactin and salmochelin (catecholate type), aerobactin (citrate-hydroxamate type), and yersiniabactin (phenolate type) [10,11]. The strength of Fe (III) binding to the molecule depends on the structure of the siderophore [12]. Enterobactin (Ent) is the strongest siderophore, with high affinities for both ferric ions and siderophore receptors [13,14] and is encoded by the *ent-fes-fep* gene cluster [15]. Due to the high-affinity binding capacity of the siderophore receptors, enterobactin can be used in strategies for pathogen detection [14] and as an enterobactin-drug conjugate in antibacterial therapy [16].

Bacterial aerobactin (Aer) is encoded by the *iucDBAC* gene cluster, salmochelin (Sal) with an *iroBCDEN* gene cluster is located on the ColV plasmid, ColBM [17] or chromosomal pathogenicity island (PAI) [18,19], and yersiniabactin (Ybt) with the *irp2, irp1* and *ybtSETUXPQA* gene cluster is located in high pathogenicity islands (HPI) [20].

The urinary iron content of healthy people is usually very low (~0.1 μmol/L) [21]. While urinary iron levels increase slightly during infection, Fe bioavailability may be limited by neutrophil gelatinase-associated lipocalin (NGAL), also called lipocalin-2 (Lcn2) or siderocalin (Scn) [22–26] Lcn2 can bind to both apo- and ferric enterobactin. When Lcn2 binds to Fe(III)-Ent, it sequesters the iron and inhibits bacterial growth. However, when Lcn2 binds to apo-enterobactin, it only sequesters the enterobactin and not the iron, which can still interfere with the uptake of iron by the bacteria and ultimately limit the growth of the bacteria [27]. Bacterial strains producing uniquely Ent cause inflammation if NGAL is deficient [21,28]. It should be noted that in the kidney, lipocalin-2-mediated iron transport is necessary to protect of the kidney from damage [29,30]. Srinivasan et al., (2012) found that lipocalin-2 deficiency leads to dysregulation of iron homeostasis and the exacerbation of endotoxin-induced sepsis [31]. Lcn2 does not affect another chemical type of siderophores (e.g., Sal, Ybt or Aer). UPEC strains have been observed to have a higher number of Lcn2-resistant siderophores, giving them a better chance of survival in the urinary tract. The Ybt and Aer siderophores are critical in *E. coli* cystitis [25]. Moreover, Ybt and Sal are the most common non-enterobactin siderophores associated with UTI recurrence [32].

Apart from siderophores, other systems are also involved in iron uptake, for example, Iha protein (homologous to IrgA) encoded by the *iha* gene. According to Léveillé et al. (2006), Iha may also function as a catecholate siderophore receptor in UTI strains [33].

The ferric citrate uptake system is another essential iron acquisition system that is highly enriched in UPEC strains [34]. Ferric citrate acts as an inducer for the transcription of the *fecABCDE* transport genes, which encode the ferric citrate transport proteins [35,36]. The binding of ferric citrate to FecA is sufficient to initiate transcription without further transport into the periplasm [35].

Approximately 70% of iron is bound to hemoglobin, making heme the most abundant source of iron in the human body. The Chu system imports heme and is more abundant in UPEC than commensal [18]. Hagan et al., (2009) revealed that the *chuA* gene expression is highly elevated in the UPEC strains that cause recurrent infections [37]. Thus, heme importers appear to play an important role in the pathogenesis of UPEC-dependent UTI [37].

Also, toxins help the pathogen to spread into deeper tissues after disrupting cell integrity; they destroy effector immune cells and thereby circumvent their potential antibacterial activity. HlyA toxin (α -hemolysin) has lytic activity against erythrocytes and interacts with natural killer (NK) cells [38].

Reduced iron availability after an infection has been observed, which may be a host defense mechanism against pathogenic microorganisms [39]. *E. coli* is also involved in protein-bound sources of iron from transferrin or heme. Thus, the typical host response to bacterial infection is the down-regulation of iron-binding proteins such as lactoferrin or transferrin.

A change in the environmental niche can induce various changes at the level of the transcription and protein synthesis of bacteria. It has been shown that the transcriptomic and proteomic profiles change under several different circumstances, such as the growth phase of the cell, oxidative stress, pH variation, or starvation [40,41]. Analyzing the proteome of bacterial pathogens such as *E. coli* holds immense significance in understanding the molecular basis of infectious diseases, particularly UTIs and urosepsis [42]. The proteomic analysis of *E. coli* from UTI and urosepsis samples can reveal distinctive protein profiles [43] shedding light on key Vfs, antibiotic resistance mechanisms, and potential therapeutic targets. In the study of *E. coli*, proteins vary between strains, and this, in turn, influences pathogenicity.

Sepsis is a severe clinical condition related to the host’s response to infection and may develop from a UTI due to an activation cascade that will lead to amplifying cytokine production, namely the cytokine storm [44,45] Interleukins include various proteins (pro-, e.g., IL-6 and anti-inflammatory, e.g., IL-4, IL-10) secreted by leukocytes and endothelial cells that contribute to cell signalling and stimulate the activation, proliferation, death or motility of immune cells [46]. A better understanding of the relationships between protein and cytokine expression profiles and finding which factors may trigger the cytokine cascade could be crucial in differentiating sepsis from a UTI [47].

C-reactive Protein (CRP) plays a crucial role as an acute-phase protein produced by the liver in response to inflammation, infection, or tissue injury. In the context of urosepsis, a severe condition arising from UTIs, CRP serves as a pivotal biomarker. Elevated CRP levels in urosepsis may correlate with a more pronounced inflammatory response and could serve as a prognostic indicator. The positive association observed between CRP, serum ferritin, IL-6 and tissue necrosis factor-alpha (TNF) underscores its role within a broader network of inflammatory markers [48].

We hypothesized that some *E. coli* siderophores are involved in bacterial adaptation in the bloodstream of UTI patients, leading to urosepsis. This study focuses on characterizing the iron uptake system of UPEC strains that cause urosepsis and investigating the expression levels of genes encoding siderophores in urine and blood. We present a comprehensive exploration of the unique protein composition in *E. coli* isolated from UTI and urosepsis cases, unraveling the molecular intricacies that underlie these critical bacterial infections, and describing a stepping-stone towards more effective diagnostic and therapeutic interventions. In addition to bacterial factors, pro-inflammatory factors of host are also important in sepsis, so we also analyzed the patients’ blood in response to infection with UTI strains.

## Materials and methods

### Settings of the clinical part of the study

All patients admitted to the Emergency Department (ED) of the Medical University of Gdansk between 2019 and 2022 underwent triage based on the Manchester Triage System. Triage is based upon measurements of the patient’s vital signs (including heart rate, blood pressure, core body temperature, respiratory rate, blood oxygen saturation, and level of consciousness measured with the Glasgow Coma Scale). These measurements allow us to quantify the clinical items of SIRS (systemic inflammatory response syndrome). The staff of the ED follow a Standardized Operational Procedure (SOP) dedicated to patients with suspicion of sepsis, which aims at the rapid identification of subjects with sepsis or septic shock and initiation of the therapeutic process. Medical history was taken from all participating subjects and all of them underwent a physical examination performed by emergency medicine specialists with experience in sepsis diagnosis and treatment.

### Patient flow and inclusion

All patients with clinical suspicion of sepsis, defined as the presence of two out of four clinical items of SIRS noted during triage, were initially included in the study.

A standardized set of laboratory tests and radiological assessments was performed, which included: arterial blood gas analysis, concentration of lactate, bilirubin, creatinine, sodium, calcium, C-reactive protein, complete peripheral blood count, procalcitonin, coagulogram and chest X-ray.

Data from medical history, clinical examination, and results of the tests of the diagnosis of sepsis were obtained according to the Surviving Sepsis Campaign guidelines (https://www.sccm.org/Clinical-Resources/Guidelines/Guidelines/Surviving-Sepsis-Guidelines-2021; Critical Care Medicine: October 4, 2021), with a decrease in Sequential Organ Failure Assessment (SOFA) Score equal to or greater than 2 as the cut-off value.

Our study groups were patients with uncomplicated UTI (acute bacterial infection of the bladder and acute uncomplicated pyelonephritis). They have no structural abnormality of the urinary tract and no comorbidities such as diabetes, an immunocompromised state, recent urologic surgery, or pregnancy). There were no patients with asymptomatic bacteriuria. Patients with a diagnosis of sepsis and UTI were included in the research group, and patients with UTI but without sepsis were included in the control group (total 450 patients).

The further selection of patients was made in relation to the final diagnosis and establishing the causative factor. Patients diagnosed with sepsis as a result of a urinary tract infection caused by *E. coli* were included in the study group, and patients without sepsis with a urinary tract infection caused by *E. coli* were included in the control group.

All procedures in studies involving human subjects were conducted in accordance with the ethical standards of the institutional and/or national research committee and with the Helsinki Declaration of 1964 and its subsequent amendments or equivalent ethical standards. The study was approved by a local Bioethical Committee of the Medical University of Gdansk (NKBBN/133/2019), and each patient gave written informed consent. The recruitment period of samples for the purposes of this study: START: 14/MARCH/2019/ End: 31/MARCH/ 2022 in the Emergency Department (ED) of the Medical University of Gdansk. Recruiting patients was part of standard clinical/laboratory practice. The study did not include minors. Technical staff collected and archived clinical isolates according to hospital procedures; researchers had no access to information that could identify individual participants during data collection.

### Samples and isolates

Urine cultures, together with aerobic and anaerobic blood cultures, were ordered for each patient enrolled in the study (samples were collected up to 72 hours after presentation at the Emergency Department). A MALDI-TOF Biotyper 117 instrument (Bruker, Karlsruhe, Germany) was used for bacterial identification. *E. coli* cultures were processed by the Clinical Bacteriology Laboratory and stored in deep-frozen (−80°C) cultures (CryobankTM, MAST Group Ltd., Germany) before the evaluation of the batches for bacterial characteristics.

Finally, the patients were divided into two groups: patients with urosepsis due to *E. coli* (Pn = 64: 32 women and 32 men; age of patients: 24–80; 384 isolates – 3 isolates from blood and 3 isolates from urine per patient) and patients with UTI due to *E. coli*, no sepsis – as a control group (Pn = 85: 63 women and 22 men; age of patients: 20–81; 85 isolates from urine). Simultaneously, additional samples of blood and urine for further proteomic and immunochemistry study-related analysis were collected and stored in −80ºC.

The main scheme of the research is shown in S1 Fig in S1 File. The procedure for obtaining research strains from patients with suspected urosepsis is shown in S2 Fig in S1 File.

### DNA extraction

*E. coli* genomic DNA was extracted using the GENOMIC DNA KIT (BLIRT S.A., Gdansk, Poland) according to the manufacturer’s protocol. The extracted DNA concentration was measured using a NanoDrop ND-100 (Thermo Fisher Scientific, Wilmington, USA) and ranged from 20 to 60 ng/µl.

### Genotyping of isolates

Additionally, the exclusion criteria were suspicion or diagnosis of hospital infection. A minimum of 3 blood colonies and 3 urine colonies per patient were tested to monitor the spread of bacteria within patients to confirm urosepsis and exclude an exogenous source of infection. For confirmation of urosepsis, *E. coli* isolates from the blood and urine of each patient with clinical suspicion of sepsis were tested using the PCR MP (PCR melting profile) fingerprinting method [49]. A total of 384 isolates in the urosepsis group were tested. DNA fingerprinting patterns were separated by polyacrylamide gel electrophoresis (6%) in 1x TBE buffer and stained with ethidium bromide. Images of the gels were analyzed and archived using the UVITEC CAMBRIDGE (FIREREADER V10; United Kingdom). We considered samples clonally related if they displayed the same electrophoretic patterns or with slight variations in the pattern up to 3 bands (a similarity coefficient (Sc) of 1–0.86) [50].

The unweighted pair group method with mathematical averaging (UPGMA) was used for cluster analysis of DNA relatedness for epidemiological study. CLIQS 1D image analysis software, version 1.5.169, was used to convert and analyze the patterns obtained from the electropherogram.

### PCR assay for the detection of iron-uptake system genes

All primers’ sequences and sizes of amplicons are provided in S1 Table in S1 File.

*E. coli* strains CFT073 (ATCC 700928), J96 (ATCC 700336) and clinical strains from our previous research (the collection of the Gdansk University of Technology) were used as positive controls. All amplification products were detected on a 1.5% agarose gel with ethidium bromide. Samples were electrophoretically separated in a 0.5x TBE buffer for approximately 40 minutes at 100V. The gel was then analyzed under UV using the Uvitec Cambridge system and the UVITec1D program.

#### PCR detection of siderophore-associated genes.

Two multiplex PCR-based methods were used to determine the presence of eight siderophore genes (enterobactin: receptor gene – *fepA*, synthesis gene – *entB*, aerobactin: receptor gene – *iutA,* synthesis gene – *iucA*; salmochelin: receptor gene – *iroN,* synthesis gene – *iroB;* yersinobactin: receptor gene – *fyuA*, synthesis gene – *irp2*). PCR reactions were performed in a 25 μl final volume with Taq DNA polymerase (2 U μl − 1) (Taq Nova BLIRT S.A. DNA Gdańsk, POLAND) in a buffer with (NH_4_)_2_SO_4_, and 0.2 mM of each dNTP, 2 mM of 50 mM MgCl_2_ and 0.4 μM of the primers. Multiplex PCR/I for the genes *fyuA*, *irp2*, *iroN*, *iroB* and multiplex PCR/II for the genes *fepA*, *entB*, *iutA* and *iucA* were assayed using the following protocol: initial denaturation 95.0°С–180 s; 30 cycles: denaturation 95.0°С – 30 s, primers annealing 54.4⁰C – 30 s and 53⁰C – 30 s, respectively, for multiplex PCR/I and multiplex PCR/II, elongation 72.0°С – 30 s; and final extension at 72.0°С – 60 s.

#### PCR detection of other genes involved in accessing iron.

In addition to siderophores, the *hlyA, iha, fecA* and *chuA* genes were detected by simplex PCR. The PCR program was used: initial denaturation 95.0°С – 180 s; 30 cycles: denaturation 95.0°С – 30 s, primers annealing – 63^o^C for *hly* and *iha*, 60^o^C for *fecA*, and 55^o^C for *chuA* – 30 s; elongation 72.0°С – 30 s; and final extension at 72.0°С – 60 s). For all reactions, we used Taq DNA polymerase (Taq Nova BLIRT S.A. DNA Gdańsk, Poland).

### Activity testing of siderophores

#### Selection of the bacterial growth phase for the study of siderophore gene expression levels.

**Fig 1:**
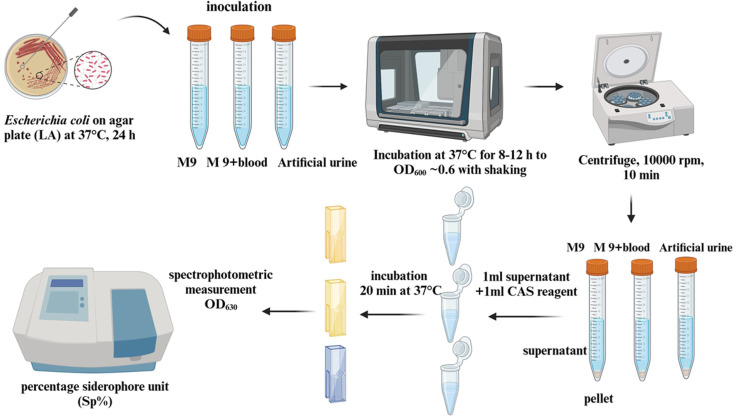
Study of siderophore activity using the CAS assay under different conditions of cultivation. Figure is created in BioRender (Licence RZ26QPUF3K).

In the course of the experiments carried out, the differences in the expression levels of the siderophore genes were examined for the selected 10 *E. coli* isolates of 5 urosepsis patients with 4 detected siderophore genes (*irp2* + *iroB +* *entC* + *iucA*) divided into groups: isolates from urine vs. isolates from blood; isolates growing on M9 minimal medium vs. isolates growing on artificial urine; isolates from urine, growing on artificial urine: logarithmic growth phase vs. stationary phase; isolates from blood, growing on artificial urine: logarithmic growth phase vs. stationary phase. OD_600_ for the logarithmic growth phase was ~ 0.6, while for the stationary phase, it was about 1.6. The culture time was different, depending on the isolate studied. Data about the optimization of the real-time PCR experiment are included in S1 Text in S1 File with standard curves of the real-time PCR for tested genes (Table 1 and Fig 1 in S1 Text in S1 File).

#### Preparation of samples for the testing of siderophore activity.

The experiments were conducted for 29 strains isolated from the blood of patients with confirmed urosepsis (urosepsis group: U1-U29), and 15 isolates from the control group: C1-C15. Our criteria for the selection of strains were based on the different number of genes encoding siderophores and belonging to different classes and each isolate came from a different patient. In addition, the same siderophore composition (if possible) was studied from different isolates.

*E. coli* strains were incubated on a lysogeny broth agar plate (LA) at 37°C for 24 h. Next, the bacterial colonies were transferred to 1) artificial urine, 2) M9 liquid medium (Sigma), 3) M9 medium supplemented with whole blood (the blood came from a healthy blood donor from the provincial Blood Donor Station in Gdańsk, Poland).

Artificial urine was prepared according to Brooks and Keevil (1997) [51]. Components for artificial urine and the recipe for preparation artificial urine, M9 and M9 with blood are presented in S2 Text in S1 File.

#### RT-qPCR for measurements of siderophore gene expression.

Genes involved in siderophore synthesis (*entC, iroB, irp2, iucA*) were analyzed, and the reference gene *rpoB*, encoding the RNA polymerase sigma factor RpoB [52]. Primer sequences, products of PCR and the optimized annealing temperature for primers in RT-qPCR are provided in S2 Table in S1 File.

After cultivation, the samples were immediately processed in an RNA lysis buffer. Total RNA was extracted using a Total RNA Mini Plus kit (A&A Biotechnology, Poland), and a Clean-Up RNA Concentration kit (A&A Biotechnology, Poland) was used for protect the quality of RNA before nuclease and for sample compaction. For all test samples, the quality of the total RNA obtained was checked by electrophoresis, visualizing the two bands (16S and 23S RNA) in a 1.5% agarose gel in 1x TBE buffer. Besides the electrophoresis method, Nano-drop1000 (ThermoScientific) was used to estimate the purity of RNA (A260/A280 and A260/A230 with a ratio close to 2). Once extracted, RNA was stored at −80ºC until needed.

The reverse transcription was carried out according to the manufacturer’s instructions for a TranScriba reverse transcription kit (A&A Biotechnology, Gdansk, Poland). Random hexamers (A&A Biotechnology, Poland) were used as primers in the reverse transcription reaction, and each reaction was carried out with 1 μg of total RNA. The cDNA was stored at −80°C until further analysis in RT-qPCR. The cDNA was diluted 20x for real-time PCR.

RT-qPCR was prepared with the SsoAdvanced SYBR Green Supermix kit (Bio-Rad Poland Ltd.,Warsaw, Poland) according to the manufacturer’s recommendations. Primer sequences for real-time PCR and the optimal annealing temperature for a given primer pair are shown in S2 Table in S1 File.

#### Phenotypic liquid CAS-assay for the measurement of siderophore production.

The same *E. coli* strains as for the RT-qPCR were selected to measure the gene expression by CAS-assay. For the quantitative estimation of siderophores, the colorimetric method modified based on the basic Chrome Azurol S (CAS) assay was used [53]. The CAS liquid assay detects total siderophores in chemically defined media regardless of the chemical nature of the siderophore. If the medium changes color from blue to orange, this indicates the chelation of the iron-siderophore complex, which we can measure using spectrophotometry.

Three media were used to cultivate bacteria from the urosepsis and control groups (M9, M9 supplemented with human blood, and artificial urine). 10 mL of fresh media was distributed into 15 mL falcon tubes and 100 µl of overnight culture was added. All cultures were incubated at 37°C, 140 rpm for 8–12 hours to OD600 ~ 0.6. The cultures were then centrifuged at 10 000 rpm (5746 g) for 10 min (Eppendorf Microcentrifuge 5415), the pellets were discarded and the supernatants were used to estimate siderophore activity [54]. The CAS reagent necessary for spectrophotometric measurements was prepared according to Louden et al., 2011. 1 ml of the supernatant from each culture was mixed with 1 ml of the CAS reagent and then incubated for 20 min at 37^o^C. Cultures were prepared in triplicate for each trial. After incubation, absorbance was 3-fold measured at 630 nm according to controls without cultures.

Siderophore activity for all strains was expressed as percentage siderophore unit (Sp%), which was calculated using the Payne formula [55]:


Sp%=[((Ar−As))/Ar]x100

Sp% – percentage activity of siderophores; Ar – reference absorbance value (absorbance for the sample with the CAS medium and un-inoculated broth); As – averaged absorbance value for the testing sample.

The same steps were performed for all testing samples. The experimental protocol for testing siderophore activity using the CAS medium is illustrated in Fig 1.

### Detection of pro-inflammatory factors

Pro-inflammatory factors were detected and measured from human serum. Serum cytokines were tested with Cytometric Bead Array Human Th1/Th2 Cytokine Kit II (BD Bioscience, Franklin Lakes, NJ) strictly according to the manufacturer’s recommendations. Briefly, 50 μl of centrifuged serum samples were added to the 50 μl of beads mixture, containing IL-2, IL-4, IL-6, IL-10, TNF-α, and IFN-γ capture beads. Next, 50 μl of PE detection reagent was added, and samples were incubated in the dark for 3 hours. Later, samples were washed and resuspended in a wash buffer. The readout was done with a BD FACSVia flow cytometer (BD Bioscience, Franklin Lakes, NJ), and the results were analyzed in FCAP Array Software (BD Bioscience, Franklin Lakes, NJ). The cytokine concentration was calculated according to the human Th1/Th2 standard using the 4-parameter curve fit option from the software. Interlaboratory comparison (ILC) samples were used as an analytical control (RIQAS Cytokine Programme, UK).

Serum blood was the test for lipocalin-2 (NGAL) level. The samples were stored at −80ºC until testing. The lipocalin-2 was tested by a Human Lipocalin-2/NGAL Quantikine ELISA reagent kit from R&D Systems (Minneapolis, MN, USA) with an assay range of 0.2–10 ng/mL and a sensitivity of 0.04 ng/mL. The readout was done with Biotek Epoch Microplate Reader (Agilent, Santa Clara, CA). For the analytical control, Quantikine Immunoassay Control Set 1076 for Human Lipocalin-2 was used (R&D Systems, Minneapolis, MN, USA).

### Proteomic analysis

Non-targeted (shotgun) proteomic analysis was performed on *E. coli* isolates (control and urosepsis groups) in artificial urine [56–59]. The artificial urine was modified for the proteomic study (data in S3 Text in S1 File). A sterile liquid medium (10 mL) was inoculated with rejuvenated bacteria obtained from solid cultures of E. coli strains. The culture of *E. coli* was incubated for 8−12 h at 37°C with continuous shaking at 140 rpm to OD600 ~ 0.4–0.6. The optical density (OD600) of bacterial cultures for proteomic profiles is given in S3 Table in S1 File. The preparation of the matrix for the proteomic studies was carried out according to Walters et al., (2009) [60], and Aggarwal et al., (2006) [61] with some modifications.

After incubation, the culture was centrifuged (4000xg, 10 min, 4oC). Afterwards, the metabolism was quickly quenched by adding cold methanol solution to the supernatant (1/20, v/v, −60°C), and the solutions were merged after incubation. For LC-MS analysis, only the supernatant was used for proteomic analysis. We separated the cell from a liquid to monitor only the extracellular proteins. After the initial sample preparation, 5 µl of protein solution was transferred to an Eppendorf tube, then 40 µl of 63 mM ammonium bicarbonate buffer, 2 µl of 2 mM DTT (dithiothreitol), 5 µl of 40 mM iodoacetamide and 3 µl of trypsin solution were added. The Eppendorf tubes were placed in a thermal shaker at 16°C with a shaking speed of 700 rpm for 16 hours. After incubation, the samples were concentrated using vacuum evaporation (3h, 40°C), followed by the addition of 50 µl of 2% formic acid solution. Next, 30 μl of each digested sample was transferred to inserts placed in LC vials. The vials were then placed in a shaker and mixed for 5 min. Finally, the prepared solutions were subjected to LC-MS analysis.

The supernatant was lyophilized, and the powder was resuspended in 50 μL of 0.1% formic acid. This solution (1 µl) was injected into a Thermo Scientific UltiMate 3000 (Thermo Scientific, USA) nano-LC system connected online with a tripleTOF apparatus (Sciex, Canada). The trap cartridges (length 5 mm, ID 300 μm), packed with C18, 5 μm PepMap100 sorbent (Thermo Fisher Scientific, USA) were used to concentrate and desalt the non-digested samples, using a 10 μl/min flow of a loading buffer (2% ACN, 0.1% TFA, H_2_O) for 3 min. Peptides were separated on a 75 μm x 250 mm fused-silica analytical column packed with PepMap 2 μm sorbent (Thermo Scientific, USA). The analytical gradient was performed by a linear increase of the mobile phase B (0.1% FA in 80% ACN) in the mobile phase A (0.1% FA in water), from 2% to 40% B in 20 min, then to 99% B during 40 min, with a flow rate of 300 nl/min. The column oven temperature was set to 35°C. Peptides eluting from the column were ionized in an OtpiFlow nano-ion source (ESI) and introduced to a SCIEX TT6600 + mass spectrometer at positive ionization mode. MS operation parameters were as follows: the spray voltage was + 5.5 kV, the nebulizer gas (N_2_) pressure was 14 psi, the collision energy was + 10 V, the declustering potential was + 90 V, and the source temperature was 210°C. The full-scan range was set at m/z 350–1500. Each full scan was followed by fragmentation of the top 10 most intense precursor ions with a charge from +2 to +5. The collision energy was from 25 to 50 eV, with a 10 eV spread. We used Analyst v.1.8 (Sciex, Canada) for data collection and PeakView 2.2 (Sciex, Canada) for data visualization.

### Statistical analyses and software

Fisher’s exact test was used to compare the proportions of Vfs. The probability threshold P-value was assumed to be at the level of ≤0.05. Logistic regression analyses were performed to find siderophores and other iron (Fe cation) uptake mechanisms and their ability to act as a risk factor for urosepsis. We also created Venn diagrams for the co-occurrence of the biosynthesis of siderophore genes using Inkscape software (Brooklyn, New York, NY). UpSet plots were generated using R script with UpSetR package (v 1.4.0) to visualize the distribution and coexistence of genes encoding factors involved in receiving and accessing iron uptake.

Significance of changes in relative gene expression in *E. coli* isolates at different growth phases and to compare relative gene expression between cases with urosepsis and controls was assessed by the Mann-Whitney U test. For analyses of relative gene expression after a change of medium either along cases or controls (3 comparisons) the Friedman’s test for paired samples was applied. The statistical analyses were performed in XLSTAT (Addinsoft). The significance level was set at P ≤ 0.05. Peptides were identified by use of ProteinPilot software with the Paragon algorithm (https://sciex.com/products/software/proteinpilot-software). The software identified between 10 000 and 11 000 MS/MS spectra (~96% of the total), ~ 6 000 distinct peptides, which resulted in ~700 proteins detected with at least 2 peptides at a 95% confidence level. The proteins marked as present were at least 100 cps for the respective peptide peak area, and the proteins marked as elevated were at least 20% higher in the peak area.

### Sample size

To determine the required sample size in this study, the XLStat software by Addinsoft, Life Science version 202.1.1 was applied. Sample size which allows to determine difference in means: mean 1 = 100 units (StDev = 35) and mean 2 = 120 units (StDev = 35), with power parameter (1-ß)=0.80 and α = 0.05, was 114 samples – 57 samples in each group, Our study consists of 64 patients with urosepsis (cases) and 85 patients with UTI (control group). Given this size of groups, the significance of difference in means (α = 0.05), as described above, is calculated with power (1-ß)=0.89. The difference in proportion of tested parameter (e.g., gene presence) equal 0.5 (group 1) and 0.75 (group 2), for an α = 0.05 and our sample size, is assessed with the power (1-ß)=0,885.

## Results

### Selection of strains responsible for urosepsis

The confirmation of urosepsis by PCR MP (PCR melting profile) genetic typing of bacterial strains isolated from the urine and blood of patients with symptoms of sepsis was carried out. PCR MP has a high discriminatory power and is used in epidemiology studies [49,62,63]. Indistinguishable isolates (the same genotype) and subtypes of the same genotype from one patient were found to be related and responsible for urosepsis due to UTI. Isolates from urine marked as identical to blood suggested translocation from the urinary system to the bloodstream and confirmed urosepsis (S3 Fig in S1 File).

All sepsis isolates were also analyzed for possible hospital-acquired infections. The genotyping by PCR-MP showed a high level of genetic diversity among the strains, suggesting that none of these strains were a nosocomial infection (S4 Fig in S1 File).

### Distribution of siderophore genes and other factors involved in receiving and accessing iron

The distribution of siderophore genes of *E. coli* strains from the control group and the UTI-dependent urosepsis group was investigated (Table 1).

**Table 1:** Comparison of bacterial virulence genes encoding iron-uptake systems in *E. coli* isolate derivates from UTI (control group) and urosepsis.

System for uptake Fe	Genes	Control group n = 85 (%)	Urosepsis group n = 64 (%)	P*
Enterobactin	*entB*	82 (96%)	61 (95%)	1.0
	*fepA*	82 (96%)	62 (97%)	1.0
Salmochelin	*iroB*	56 (66%)	34 (53%)	0.13
	*iroN*	54 (64%)	35 (55%)	0.31
Aerobactin	***iucA***	**41 (48%)**	**43 (67%)**	**0.030**
	***iutA***	**44 (52%)**	**45 (70%)**	**0.028**
Yersiniabactin	*irp-2*	68 (80%)	56 (88%)	0.27
	*fyuA*	68 (80%)	56 (88%)	0.27
Additional iron uptake system and support	***hlyA***	**8 (9%)**	**14 (22%)**	**0.038**
	*chuA*	64 (75%)	48 (75%)	1.00
	***iha***	**17 (20%)**	**28 (44%)**	**0.002**
	*fecA*	57 (67%)	43 (67%)	1.00

Legend: *The Fisher exact test was used to compare proportions. A P ≤ 0.05 was considered statistically significant.

The one-dimensional analysis carried out on strains from the control and urosepsis groups shows that enterobactin (*entB, fepA*) was the most frequently occurring siderophore (95−97%). Yersiniabactin (*irp-2, fyuA*) was also found to be common in both groups, with a slight increase in strains isolated from urosepsis samples (80% and 88%, respectively). The aerobactin genes (*iucA, iutA*; 67% and 70%, respectively) occurred much more frequently in the urosepsis group compared to the control group (48% and 52%, respectively), and this difference was statistically significant (P < 0.05 for *iucA*; P < 0.05 for *iutA*).

Most strains were observed with three and four siderophores, although a different composition of predominant siderophores was detected in the control and the urosepsis groups. The co-dominant siderophore composition of “enterobactin + salmochelin + yersiniabactin” was more frequent in strains isolated from the control group (P < 0.05), while the siderophore composition of “enterobactin + aerobactin + yersiniabactin” was typical for strains isolated from the urosepsis group (P < 0.05) (S5 Fig and S4 Table in S1 File).

In addition, the *fecA* gene was detected in 67% of the samples from both groups. This result highlights that the ferric citrate uptake system is an important iron uptake system in UTI and urosepsis strains. Moreover, an additional iron uptake system deserves special attention, the catecholate siderophore receptor Iha, which twice as frequently appears in sepsis isolates (P < 0.05). The presence of the *hlyA* gene (P < 0.05) may also favour the development of sepsis and is a risk factor for death.

**Fig 2:**
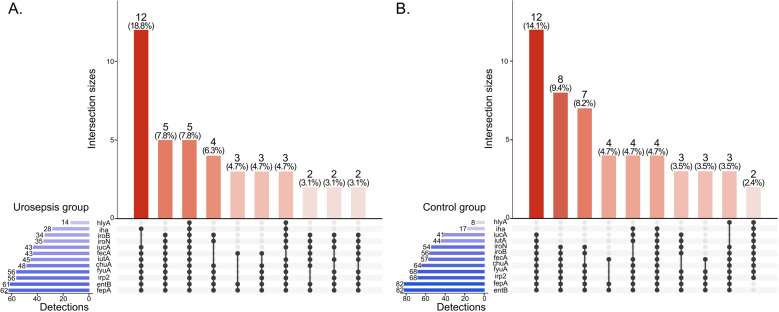
UpSet plots with distribution and coexistence of genes encoding factors involved in receiving and accessing iron uptake. A – urosepsis group (n = 64), and B – control group (n = 85). Legend: *chuA* - hemin receptor; *entB-* synthesis enterobactin; *fecA*- outer membrane receptor – binding of ferric citrate; *fepA* –ferric enterobactin receptor; *fyuA* –ferric yersiniabactin receptor; *iha* -bifunctional enterobactin receptor/adhesin protein; *iroB* - synthesis salmochelin; *iroN* - ferric salmochelin receptor; *irp-2* – synthesis yersiniabactin; *iucA*- synthesis aerobactin; *iutA* - ferric aerobactin receptor. UpSet plots were generated using R script with UpSetR package (v 1.4.0).

Distribution and coexistence of iron uptake system and additional support for iron is presented in Fig 2.

In the case of the urosepsis group, 7.8% of the isolates had all the genes tested that are related to iron uptake, whereas in the control group there was no strain with all the genes tested. The most numerous were urosepsis strains (18.8%) for which only *iroB, iroN* (salmochelin siderophore) and *hlyA* gene encoding hemolysin were missing. The remaining genes of other siderophores and additional iron uptake system and support were present.

### Expression of siderophore synthesis-associated genes

#### Study of relative expression of siderophore biosynthesis genes regarding the growth phase for urosepsis isolates.

**Fig 3:**
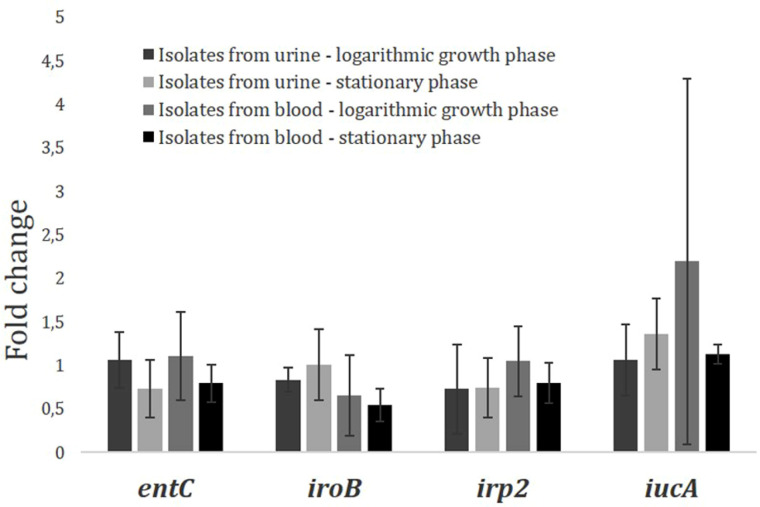
Changes in the relative expression levels of the siderophore biosynthesis genes studied from *E. coli* isolates obtained from patients’ urine and blood during culture on artificial urine. Results are presented for their growth at the logarithmic and stationary phase. The bars represent the mean value of the relative expression level of the *entC, iroB, irp2, iucA genes*; error bars represent the minimum and maximum value.

**Fig 4:**
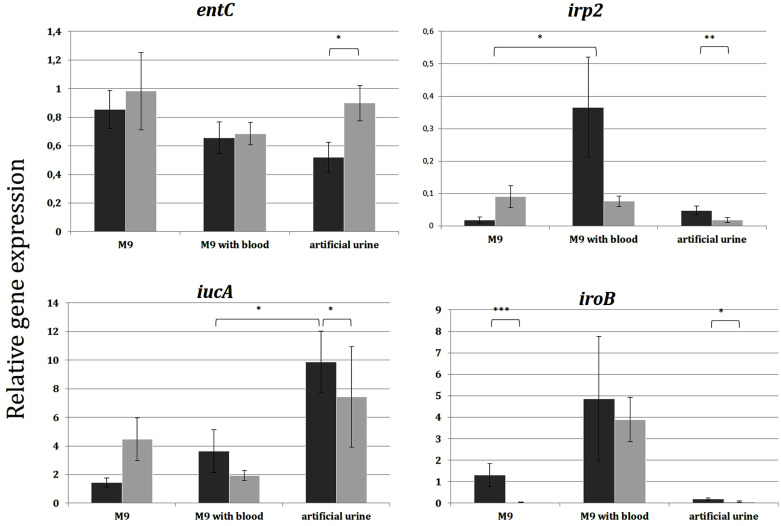
Comparison of the expression of siderophore biosynthesis-associated genes *entC* (enterobactin), *irp2* (yersiniabactin), *iucA* (aerobactin), *iroB* (salmochelin) with RT-qPCR. The sample sizes were n = 15 for the control group and n = 29 for urosepsis. Legend: *P ≤ 0.05 ** P < 0.01; ***P < 0.0001. The statistical significance was determined using the Friedman and Wilcoxon tests. Urosepsis group – light grey color; control group – dark grey color.

To show whether the expression levels of genes involved in siderophore biosynthesis differ with regards to the growth phase of bacteria the relative expression levels of siderophore biosynthesis genes for *E. coli* strains isolated from urine and blood of urosepsis patients were compared (Fig 1-4 in S4 Text in S1 File; Fig 1 and Table 1–3 in S5 Text in S1 File).

The averaged results of fold changes in the expression levels of the siderophore genes of *E. coli* isolates collected from both urine and blood of patients with urosepsis group, in the logarithmic growth phase and stationary growth phase, are presented in Table 2 and Fig 3.

**Table 2:** The averaged results of fold changes in the expression levels of the siderophore genes of *E. coli* isolates in logarithmic and stationary growth phases.

Artificial urine/M9 medium								
Fold change in gene expression								
Genes	*entC*	P*	*iroB*	P*	*irp2*	P*	*iucA*	P*
***E.coli* isolates from patients’ urine (n = 5)**								
**Logarithmic growth phase**	1,066	>0.05	0,835	>0.005	0,73	>0.05	1,066	**≤0.05**
**Stationary growth phase**	0,729		1,007		0,744		1,36	
***E.coli* isolates from patients’ blood (n = 5)**								
**Logarithmic growth phase**	1,11	>0.05	0,657	>0.05	1,049	>0.05	**2,195**	**<0.001**
**Stationary growth phase**	0,794		0,545		0,799		1,131	

*P ≤ 0.05 was considered statistically significant with Mann-Whitney U test. See Tables 2,3 in the S5 Text in S1 File for a detailed analysis of the comparisons between groups.

The test showed that the differences in the levels of gene expression associated with *E. coli* siderophore biosynthesis between the four groups of isolates considered (urine isolates in logarithmic growth phase, urine isolates in stationary phase, blood isolates in logarithmic growth phase and blood isolates in stationary growth phase) for patients with urosepsis were not statistically significant, except for the *iucA* gene (P ≤ 0.05). However, the highest changes in the relative expression levels of *iucA* were observed for isolates from blood in the logarithmic growth phase, hence in further research, logarithmic growth phase was selected to assess gene expression levels for both the control and urosepsis groups.

#### Expression of siderophore biosynthesis-associated genes with RT-qPCR – urosepsis vs. control group.

The level of the *entC, iroB, irp2* and *iucA* expression has been investigated in the control group and urosepsis group under the three different culture conditions - M9 medium, artificial urine and the M9 medium supplemented with blood (S5 Table in S1 File) using RT-qPCR.

Expression levels of the *irp2* and *iucA* genes were significantly different between media (P = 0.02, P = 0.03, respectively) in the case of strains isolated from the control group but not for strains from the urosepsis group. When the *entC* expressions were compared between strains from urosepsis patients and the control group for a selected medium, they were significantly higher in strains from patients with urosepsis as compared to the control group for cultures in artificial urine (P = 0.029), while the expressions of *irp2*, *iucA* and *iroB* were significantly lower in strains from urosepsis patients in artificial urine cultures (P = 0.007, P = 0.030, P = 0.012, respectively). Furthermore, the expression of *iroB* was significantly lower in strains from urosepsis patients when compared to controls grown in M9 medium (P = 0.001) (Fig 4).

#### CAS assay.

**Fig 5:**
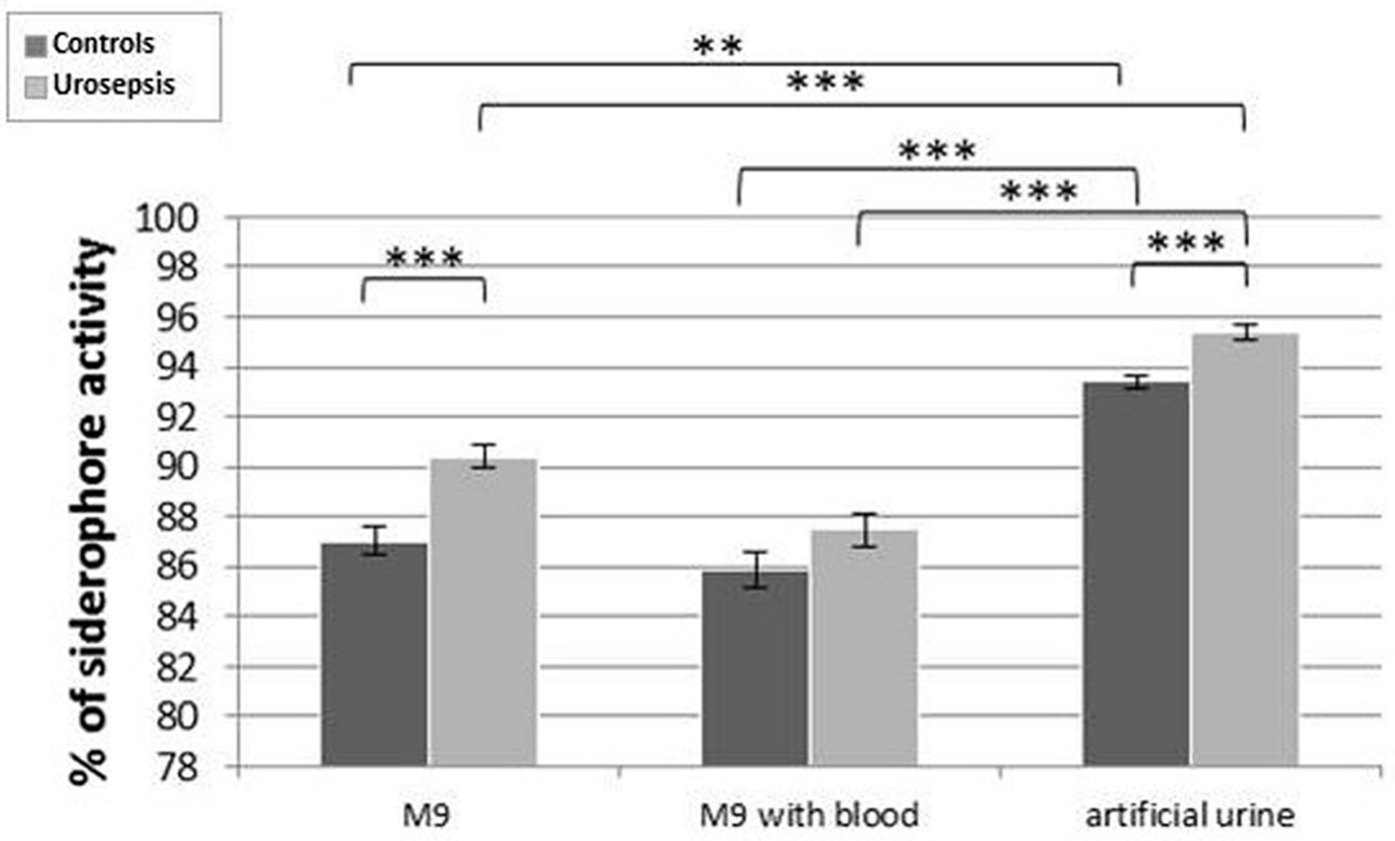
Comparison of siderophore production on three different media: M9, M9 supplemented with human blood, and artificial urine for the control and urosepsis patient groups. Legend: Light grey color – isolates from urosepsis; dark grey color – isolates from the control group. The graph shows the averaged results, including the standard error of measurement (SEM). The statistical significance was determined using the Friedman and Wilcoxon tests. Urosepsis group – light grey color; control group – dark grey color. Sample numbers were n = 15 for UTI and n = 29 for urosepsis. Legend: ** P < 0.01; ***P < 0.0001. For details, see S7 Table in S1 File.

Strains with different siderophore profiles for the control and urosepsis groups were selected for the expression study in different media (M9, M9 supplemented with whole blood and artificial urine) (S6 Table in S1 File) by CAS assay. The CAS assay is a colorimetric method and the percentage activity of siderophores (Sp%) was determined by spectrophotometric measurement. Isolates with 3 and 4 siderophore types were dominant in both groups, but the number of siderophore genes and type of siderophore did not affect their individual expression level. The situation changed according to the environment and the origin. The strongest expression of siderophore genes was observed in artificial urine for both groups, then in M9 with blood and M9 medium (Fig 5).

Statistically significant differences were observed in different environments for the urosepsis group (M9 vs. artificial urine P < 0.0001; M9 + blood vs. artificial urine P < 0.0001) and for the control group (M9 vs artificial urine P < 0,002; M9 + blood vs artificial urine P < 0.0001). The P-value for the expression of siderophores was also statistically significant between groups for cultivated isolates in artificial urine (P < 0.0001).

### Detection of pro-inflammatory factors

The pro-inflammatory factors IL-2,4,6,8,10 and IFN-gamma, TNF-alpha and CRP were quantified (Table 3).

**Table 3:** Pro-inflammatory factors for the urosepsis and control groups.

Pro-inflammatory factor	Average concentration [ng/ml] Urosepsis group	Average concentration [ng/ml] Control group	Median Urosepsis group	Median Control group
**IFN-gamma**	3.85 ± 5.57	2.24 ± 3.87	1.19	0.89
**TNF-alpha**	2.95 ± 6.72	1.21 ± 0.98	0.57	0.69
**IL-2**	0.57 ± 0.42	0.43 ± 0.75	0.53	0.11
**IL-4**	0.29 ± 0.09	0.31 ± 0.15	0.29	0.33
**IL-6**	669.73 ± 185.08	225 ± 465.98	**220.24**	**65.86**
**IL-8**	216.11 ± 352.36	47.04 ± 82.58	**67.03**	**18.53**
**IL-10**	119.78 ± 160.89	15.64 ± 17.43	**49.27**	**7.915**
**CRP**	216.92 ± 111.98	141.08 ± 106.33	**195.5**	**140.0**
**NGAL**	2.085 ± 2.24	4.37 ± 13.46	1.48	1.95

Legend: the symbol “±”, indicates the precision of an approximation.

We observed a positive association between serum ferritin, IL-6, tissue necrosis factor-alpha [TNF] and high-sensitivity C-reactive protein (CRP). In response to infection and tissue damage, NGAL protein is released by activated neutrophils. The average concentration of NGAL in blood serum was 2.085 ± 2.24 (ng/mL) in the urosepsis group and 4.37 ± 13.46 (ng/mL) for the control group. Renal injury was not detected in any of the patients, so the cause of sepsis due to renal damage was excluded. Median NGAL levels were higher in the control group than in the urosepsis group. It can indicate weak response in urosepsis patients and give a higher chance of bacterial survival.

### Proteomic LC-MS analysis

After cultivation *E. coli* in artificial urine levels of proteins were compared between *E. coli* responsible for UTI (control group) and urosepsis with the use of proteomics. Non-target proteomics and other non-siderophore proteins were detected, important in iron management.

S8 Table in S1 File summarizes the proteins that differentiate the two groups of strains that we have studied in this paper and that could be related to UTI/urosepsis states. We focused mainly on proteins related to iron transport. These are: ferritin-1, the iron uptake system component EfeO, the ferrous iron transport protein B, the nitrate/nitrite response regulator protein NarL, the protein HemY, the ferrienterobactin receptor, the lipopolysaccharide export system protein LptA and 2Fe-2S ferredoxin.

## Discussion

Urosepsis results from a clinically severe infection of the urinary tract and/or male genital tract (e.g., prostate) and accounts for approximately 25% of all sepsis cases [64]. Uropathogenic *E. coli* are the major pathogens of UTIs, which may be responsible for up to 75–85% of uncomplicated, community-acquired UTIs [65,66].

Iron is an essential nutrient for humans as well as for pathogenic bacteria and bacteria have developed a variety of effective strategies to take up iron from their environment, for example by siderophores, that scavenging ferric iron (III) [67]. Siderophores and other factors acquiring Fe (III) enhance the virulence and fitness of bacteria and may influence the development of urinary tract infections and probably sepsis.

The study aimed to answer the following questions: (1) Which groups of siderophores are dominant in urosepsis-causing strains? (2) Which siderophores show increased expression in the blood and which in the urine? (3) Is there a bacterial protein associated with iron uptake that can be a marker and predict the risk of urosepsis in urinary tract infections? (4) Is there a difference in the levels of pro-inflammatory markers between UTI and urosepsis patients?

Our study only included a group of patients with urosepsis due to community-acquired UTI. Bacterial colonization of the kidney and subsequent tissue damage increases the risk of sepsis [68].

Genetic typing using the PCR-MP method made it possible to determine the degree of relatedness between the isolated strains. By comparing the DNA fingerprint patterns of the blood and urine isolates, we were able to identify the cause of the sepsis as a UTI (S3 Fig in S1 File). Moreover, genetic typing also excluded patients with nosocomial infections (S4 Fig in S1 File).

### Fe uptake system from UTI and urosepsis isolates

Pathogenic bacteria living in the human body must survive in an environment where iron is bound to proteins such as hemoglobin, transferrin, lactoferrin and ferritin. Free iron is the most dangerous form of iron for humans, as it can cause oxidative damage to organs, but it is very beneficial for bacteria. A bacterial cell needs 10⁵ to 10⁶ iron ions per cell per generation to maintain the required internal concentration of 10 ⁻ ⁶ M [18,68].

Under such conditions, effective siderophores play a major role in bacterial survival. Siderophores are synthesized and secreted by bacteria as a means of Fe (III) acquisition when their availability is limited. The biosynthesis and acquisition of siderophores are not abilities that are possessed by all bacteria to the same extent. These molecules differ in structure, pathway of synthesis and route of transport.

The catecholate siderophore, enterobactin, is the most common siderophore of *E. coli* and *Salmonella* [18,69]. It is known that Ent exhibits both the highest affinity for ferric iron among siderophores and competes with the host iron-binding protein transferrin [70]. We found Ent was the most widely disseminated siderophore in both the control and urosepsis groups, but it was not the only iron uptake system in these bacteria. Only a small percentage of the strains (5.0% for the urosepsis group and 9% for the control group, P > 0.05) were limited to one Ent siderophore. Such an arrangement is not favorable for the bacteria. Activated Lcn2, can bind to Fe(III)-Ent complex. A neutralized siderophore limits bacteria’ access to iron and thus prevents their growth, hence Lcn2 acts as a natural bacteriostatic agent [16,22]. According to Guo et al. study Lcn2 can also sequesters the enterobactin, without Fe(III) and ultimately limit the growth of the bacteria [27].

It has been demonstrated that Lcn2 accumulates in the blood and urine of patients with acute kidney injury (AKI) and the blood of patients with bacterial infections [71]. Additionally, it was shown that during acute anemia, the expression of NGAL is reduced in erythroid cells by a feedback system We conclude that patients with low Lcn2 levels (e.g., during acute anemia) are at high risk of infection even if *E. coli* strains carry only one siderophore.

A derivative of enterobactin is salmochelin (Sal), which can replace Ent [19,72]. Sal has a high affinity for ferric iron, allowing bacteria to thrive in transferrin-rich environments [70]. In our study, Sal did not occur as the sole siderophore (S1 Table in S1 File). Sal was detected together with Ent and Ybt in 27% of isolates in the control group and 16% for urosepsis for the combination (P < 0.05). Still, Sal co-occurred relatively often with other siderophores in complex (Sal + Ent + Ybt + Aer) in both study groups (27% vs. 30% for control and urosepsis groups, P > 0.05) (S4 Table in S1 File).

The next siderophore, yersiniabactin (Ybt), is located on the high-pathogenicity island (HPI) that is disseminated by horizontal gene transfer (HGT) [73,74], thus yersiniabactin genes appeared regularly, both in strains from the control group (80%) and the urosepsis group (88%). This additional siderophore significantly increases bacterial virulence, particularly as it plays an important role for efficient biofilm formation in the urinary tract, leading to recurrent infections and kidney colonization [75].

It has been proven that Aer, besides iron acquisition, enhances biofilm formation and oxidative stress resistance [76]. Aer may aid in the translocation of bacteria from the gut to the tissues and the proliferation of bacteria in the tissues, and may be an important factor in the establishment of infection [77]. We found there was a clear dominance of Aer in the *E. coli* isolates causing urosepsis (48% control group vs. 67% urosepsis group, with P = 0.03). Furthermore, Aer has been found in our research to co-occur with other siderophores such as Ent and Ybt (26%). Such a set of siderophores gives the bacteria a high chance of survival not only in the urinary tract but also in the bloodstream, where the iron concentration is higher.

In addition to typical siderophores, the *ihaA, fecA, chuA* and *hlyA* genes were detected for both groups of isolates. Each of these genes has a different way of obtaining iron for the bacteria.

The *iha* gene was detected in recurrent episodes of cystitis and is needed for a complete pyelonephritis reaction [78]. Moreover, in Johnson’s study, the *iha* gene was detected in 55% of urosepsis isolates [2]. In our study, we also saw a difference in *ihaA* between the control and urosepsis groups (P = 0.002). According to our hypothesis, the IhaA outer membrane protein with a siderophore receptor function can be a risk marker for UTI-associated urosepsis.

The other source for acquiring iron is via host proteins such as citrate, heme, hemoglobin, hemopexin or iron bound to lactoferrin and transferrin. According to Frick-Cheng et al. (2022) there are high levels of ferric citrate within the urine, during UTI (suggested level ~10 mM) [34], whereas normal levels in healthy individuals vary from 1.7 to 6.6 mM [79]. Under iron-limiting conditions, ferric citrate uptake (*fecABCE* and *fecIR*) is highly and significantly upregulated. Outer membrane protein receptors FecA can recognize a wide range of siderophores with the same similar chelating moiety. This ability allows some species to use siderophores produced by other organisms. Using a UTI mouse model, Frick-Cheng et al., (2022) showed that *fecA* is a fitness factor that is independent of enterobactin production and more highly upregulated in the absence of Ent [34]. In urine, due to the action of Lcn-2, this factor can be very important for acquiring iron. Frick-Cheng et al. (2022) also observed that citrate levels in plasma are lower than in urine (varying from 100 to 150 μM), but sufficient for robust upregulation of *fecA* [34]. The *fecA* gene is present in 67% of our isolates for both groups, demonstrating its important role in iron-poor urine. Ferric citrate uptake does not have a direct role in the appearance of sepsis, still, it can influence the existence of bacteria in the urinary tract, and it is just one of the many other mechanisms for iron acquisition by UPEC.

Another example of iron acquisition by bacteria is the membrane protein ChuA, which is responsible for heme uptake [80]. ChuA is typical of pathogenic phylogenetic groups [81]. Up to 75% of our strains (regardless of the group) had the *chuA* gene, indicating that our strains should be considered highly virulent.

The level of free heme available in the serum is too low to be a factor supporting the growth of pathogenic microorganisms. However, some bacteria can increase the availability of free hemoglobin by secreting toxins that cause erythrocyte lysis. One of the toxins is the lipoprotein HlyA, also called α-hemolysin. It was previously reported that up to 78% of UPEC isolates from pyelonephritis cases express hemolysin [82]. Croxen et al. indicated the presence of the HlyA toxin in 31–48% of strains responsible for cystitis and in 78% of strains associated with the upper urinary tract [83]. Moreover, in our previous research, we indicated the presence of the *hlyA* gene as an important Vf in different UPEC strains [63,84]. This gene has been implicated in various roles in UPEC pathogenicity, from induced nitric oxide synthase (iNOS)-mediated cell membrane perforation to host cell apoptosis [85]. Depending on the level of the toxin, HlyA either lyses erythrocytes and induces neutrophil apoptosis or promotes exfoliation of bladder epithelial cells, providing exposure to the deeper layers of the urothelium [86,87]. In addition, HlyA induces Ca^2+^ oscillations in renal epithelial cells and disrupts normal urine flow in the ileal tubules, ultimately facilitating pathogen colonization of the ureters and involvement within the renal parenchyma [87]. In our studies, contrary to reported data, the *hlyA* gene appeared infrequently, but more often in the urosepsis group (P = 0.04).

In summary, strains isolated from urosepsis were richer in different Fe-uptake genes that it can suggest both functional redundancy and niche specificity for this pathogen and ensure survival in the circulatory system [43].

### Expression of siderophores

Knowing the siderophore profiles for the individual isolates, from the control and urosepsis groups, we determined (1) study of the influence of bacterial growth phase on siderophore gene expression in artificial urine conditions, (2) the expression of individual siderophores by using RT-qPCR, and (3) the total expression level of the iron uptake system by using the CAS assay.

#### Selecting a growth phase for gene expression studies.

According to Heffernan *et al*., (2024), Ybt concentrations showed a delayed increase compared to Ent, following the growth curve and peaking during the stationary phase in UTI89 culture [88]. The authors underlined that the Ybt system functions as both a quorum sensing and a siderophore system, and this dual function may be an adaptation to environments in the host and belong to fitness within bacterial communities.

To select the appropriate phase of bacterial growth in which the expression of siderophore genes should be studied, preliminary experiments were carried out in the logarithmic growth phase and the stationary phase in artificial urine. Genes encoding Ent, Aer and Sal showed a wide range of expression levels during our experiments. For the isolates from blood in the logarithmic growth phase, the *iucA* gene was relatively expressed at a high level, whereas the Ybt level was increased in the stationary phase. However, fold change in expression was not statistically different between growth phases; thus, the growth phase did not play a significant role in gene expression. Hence, we focused exclusively on the logarithmic growth phase of bacteria to study the expression of genes involved in siderophores biosynthesis.

#### RT-qPCR.

The expression of individual genes was studied by means of RT-qPCR. Only the biosynthesis genes *entC, iroB, irp2* and *iucA* were selected.

In our study, the expression level of *entC* in the artificial urine of isolates from patients with urosepsis was significantly higher than in the control group (P = 0.029). Ent is the most abundant siderophore, giving it a greater chance of acquiring iron in the urine. It enables UPEC to better survive the host immune system by inhibiting reactive oxygen species (ROS), phagocytosis and bactericidal activity of neutrophils [89]. *iroB*, which encodes salmochelin, had the highest expression level among other biosynthesis siderophore genes in the medium with blood. Sal is a highly efficient in the presence of serum albumin, allowing bacteria to survive in the bloodstream [2,90], making the presence of this protein a risk for urosepsis.

Ybt was common in the tested isolates (80% control group vs 88% urosepsis group), and while its expression in urine was significantly different between groups (P = 0.007), it was expressed at a low level. The low expression of Ybt in urine is probably compensated by the expression of Ent or other siderophores. The expression of Ybt in blood culture is higher than in urine, but no significant differences were found between patient groups. Similar conclusions were reached by Johnson et al. (2018), but in an in vivo experiment [91]. Using mouse models and an *irp2* deletion mutant, authors demonstrated that the *irp2* gene is important in the pathogenesis of both UTI and urosepsis.

According to Garcia et al. (2011), Aer is very effective in serum iron uptake and can acquire more iron from transferrin than Ent [11]. According to our study, the aerobactin gene was significant in the urosepsis group, but its expression is likely to be important in the urinary tract, not in the blood. Probably in the blood environment, the acquisition of iron seems to occur independently of the Aer level, but the expression of aerobactin in urine may influence the intensity of UTI and, thus the course of the disease.

#### CAS assay.

The CAS assay is the universal assay for detecting siderophores and is based on the high affinity of a siderophore for ferric iron (III), and it does not consider the chemical structure of siderophores.

We found the mean siderophore activity percentage was different depending on the environment and isolates group. The maximum siderophore production was found in artificial urine, followed by M9 and M9-supplemented blood (P < 0.0001). The ferric concentration in the artificial urine is low (similar to physiological), so in our experiment the bacteria must also produce high levels of siderophores. Blood hemolysis can occur during culture, and iron is more readily available to bacteria (70% of iron is bound to hemoglobin). Most iron in heme is in the form of Fe (II), and siderophore expression is not required to such a high degree. In the human body, hemolysis of the blood can occur due to various physical, chemical and biological factors, one of which is the presence of UPEC bacteria with the *hlyA* gene. The most severe consequence of acute hemolysis is renal failure. Differences in total siderophore production between different strains within the same medium are almost imperceptible. Interestingly, Sp% did not depend on the number of siderophore genes. Presumably, the siderophore activity was adapted to the Fe demand. High intracellular Fe concentrations can be cytotoxic, bactericidal or antimicrobial [92].

### Pro-inflammatory system in sepsis

Our study demonstrated that the levels of pro-inflammatory factors (interleukins, TNF-alpha and IFN-alpha) were significantly increased in patients with urosepsis compared to the control group. A positive association was found between serum ferritin, interleukin-6,8,10 (IL-6,8,10), TNF and CRP. Other epidemiological studies have also confirmed these findings [93]. Altered iron metabolism is reflected by a high serum ferritin level, which signals disease severity and outcomes. Moreover, increased ferritin levels can be observed in inflammatory conditions, with hyperferritinemia, a significant acute-phase reaction regarded as a marker requiring therapeutic intervention. Conversely, it is suggested that increased levels of circulating ferritin may also play a critical role in inflammation by contributing to the development of the cytokine storm [94]. To the best of our knowledge, the interactions between pro-inflammatory cytokines, namely interleukins, TNF-α, IFN-γ and other proteins have not been described sufficiently in patients with urosepsis and urinary tract infections until now.

NGAL is an interesting and promising biomarker in acute and chronic inflammatory processes [95]. NGAL expression is stimulated by damaged epithelial cells [96]. Increased expression of NGAL in tissues in various states of stress is, therefore, the mechanism by which the task is to stimulate the iron-dependent defence systems. NGAL stimulates the proliferation and reconstruction of the epithelium by regulating intrarenal iron metabolism [95,96]. NGAL level can be measured in serum and urine. NGAL in serum, together with protein C and the receptor for interleukin 1, is an optimal panel for assessing the risk of organ damage, shock and mortality in patients with sepsis [30]. In clinical situations leading to organ damage, urinary NGAL concentrations may increase due to overproduction and incomplete reabsorption of filtered protein. A decrease in glomerular filtration rate in acute kidney injury may lead to a decrease in renal clearance of NGAL with subsequent accumulation of this lipocalin in blood serum.

There has not been much research into the role of NGAL in sepsis. The ability of NGAL to bind iron is extremely important in immune processes. During infection, bacteria synthesize siderophores that sequester the iron required for their growth from the extracellular space, resulting in iron deficiency, which is necessary for the normal function of cells of the innate immune response. NGAL-deficient mice have been shown to be significantly more susceptible to infection and sepsis caused by *E. coli* [30]. Clinical studies have shown that lipocalin-2 levels rise rapidly during acute bacterial infection and inflammation in mammals. In conclusion, NGAL exerts its bacteriostatic effect by binding to siderophores and interfering with iron utilization by bacterial cells – protective function, but also high level informs about acute kidney injury In our study for patients with urosepsis, Lcn-2 levels were a little bit lower (average concentration 2.085 ± 2.24 ng/mL for urosepsis group vs. average concentration 4.37 ± 13.46 ng/mL for the control group with UTI), resulting in a weaker defence against bacteria; sequestration of Fe(III)-enterobactin complex by NGAL was not as efficient. The sepsis may have been determined by two factors – a well-developed iron uptake system by the bacteria that was competitive with the host, and the second factor, the low levels of Lcn-2 in the patients, which deprived the patients of protective function.

### Sepsis-related proteomic study

Proteomics is the large-scale study of proteins present in a cell, tissue or organism, and it plays a crucial role in understanding the molecular mechanisms and functions of living systems [58,97]. By tapping into databases and tools, proteomics deepens our understanding of protein structure and function, offering potential for innovative disease interventions [57].

Among the numerous proteins isolated from *E. coli* strains from urosepsis patients, we only analyzed those pertaining to iron metabolism and transport. These included the ferrous iron transport protein B, the nitrate/nitrite response regulator protein NarL, the ferrienterobactin receptor, ferritin-1, the iron uptake system component EfeO, the protein HemY, the lipopolysaccharide export system protein LptA and 2Fe-2S ferredoxin.

#### Ferrous iron transport protein B.

Functionally, ferrous iron transport protein B plays a critical role in the uptake of ferrous iron, an essential nutrient for many cellular processes. The role of the FeoB and FeoA proteins in ferrous iron transport and their contribution to bacterial virulence has been the subject of many studies in recent years [98]. Its importance can vary between *E. coli* strains due to their adaptation to different microenvironments (in our case, the urinary tract or blood). Protein expression can be influenced by interactions with the local environment, neighbouring bacteria and host cells. According to our observations, this protein is not produced in the urinary environment. In the presence of iron, the Fe(II)-Fur complex binds to the Fur box and inhibits the transcription of the *feo* gene [99]. However, the *feoB* gene is highly expressed in the blood, hence the elevated expression profile [100].

#### Nitrate/nitrite response regulator protein NarL.

The passage of bacteria into the circulation presents some unique stressors as the environment changes dramatically, and only those bacteria with an active Nitrate/Nitrite Response Regulator Protein NarL system can survive [101]. Functionally, the NarL protein is integral to the regulation of nitrate and nitrite metabolism. These molecules are particularly relevant in anaerobic respiration and nitrogen assimilation. However, the importance of NarL for different strains might diverge due to their adaptation to distinct environmental niches. The strains causing urosepsis and UTIs likely experience different selective pressures within the host’s body, resulting in varying levels of reliance on NarL-mediated pathways. However, adaptation to various microenvironments also plays a role. UPEC strains encounter slightly different conditions within the urinary tract, including variations in pH, nutrient availability and oxygen levels. These variations may drive the differential expression of genes, including those encoding NarL. The presence or absence of the nitrate/nitrite response regulator protein NarL in different strains of *E. coli* may be due to a complex interplay of genetic, environmental and functional factors.

#### Ferrienterobactin receptor.

The presence or absence of the ferrienterobactin receptor could be the result of evolutionary gain or loss, shaped by selective pressures imposed by the host and the environment. As our understanding of bacterial biology evolves, deeper insights into why the ferrienterobactin receptor is present or absent in different bacterial strains are likely to emerge. Interactions with host cells, immune responses and competitive dynamics within the host environment can shape bacterial expression profiles [102], and therefore, certain strains may benefit from expressing the ferrienterobactin receptor, depending on the circumstances, whereas others may not.

Using a non-targeted proteomic study, we showed that FepA protein was deficiency in the control group, although 96% of the strains carried the *fepA* gene. Likely, they do not have a functional Fep receptor for enterobactin. However, bacteria can replace it with the protein Iha, which has been shown to have a dual function as an adhesin and enterobactin receptor [2,33,78,103]. Apart from FepA and Iha, IreA, Fiu (catecholate siderophore receptor) and CirA (outer membrane receptor for ferrienterochelin and colicins) may be involved as other receptors for Ent [34,104]. The FepA protein was synthesised in the urosepsis group, making it more suitable for bacterial survival.

At the beginning of the discussion, it should be noted that the proteomic analysis conducted was untargeted, allowing for a broad view of proteins involved in iron acquisition processes by the studied bacteria. Unfortunately, siderophores could not be detected, or their presence was observed only in very low concentrations, indicating that the proteomic method in this configuration was not sufficiently sensitive for the analysis of these compounds. Therefore, a more targeted approach, such as using RT-qPCR to assess the expression of genes responsible for siderophore synthesis and transport, proved to be more appropriate for accurately evaluating these mechanisms. Moreover, shotgun proteomics does not always accurately reflect mRNA levels, as the abundance of mRNA does not necessarily correlate with the detectable presence of proteins in LC-MS analyses. This discrepancy arises because not all proteins are efficiently digested and observed during LC-MS, leading to potential gaps in the proteomic data. Therefore, establishing a direct correlation between mRNA and protein levels can be challenging. In our study, RT-qPCR was conducted in parallel with proteomic analyses to provide a comprehensive overview of gene expression. However, to specifically identify and quantify these proteins, additional standards for the target proteins derived from mRNA would be necessary. Unfortunately, obtaining such standards is not always feasible or available, adding another layer of complexity to the analysis.

#### Lipopolysaccharide export system protein LptA.

LptA plays a crucial role in the export of lipopolysaccharides, essential components of the outer membrane in Gram-negative bacteria [105]. However, the importance of LptA can vary between strains due to their adaptation to unique environments. The reliance on the LptA-mediated pathway for lipopolysaccharide transport may differ between strains associated with infections or specialized ecological niches.

The presence or absence of the lipopolysaccharide export system protein LptA in different bacterial strains, particularly in contexts such as infections or different ecological niches, can be elucidated through a nuanced interplay of genetic diversity, functional adaptation and environmental factors.

#### 2Fe-2S ferredoxin.

2Fe-2S ferredoxin serves as an essential electron carrier in a wide variety of biochemical reactions, particularly those involving redox processes. However, its importance can vary between organisms and environments due to adaptations to specific niches [106]. Organisms living in particular ecological niches or undergoing certain physiological processes may have different degrees of dependence on the 2Fe-2S ferredoxin-mediated electron transfer pathway. Interactions with the environment and host organisms also have a significant influence. The expression of this protein can be shaped by interactions with the external environment, interactions with other organisms and the biochemical dynamics at play. This dynamic interplay may result in the selective advantage of expressing 2Fe-2S ferredoxin in certain contexts, likely driven by microenvironmental factors. Organisms occupying different habitats experience different nutrient availability, temperature ranges and other physical and chemical conditions. These factors contribute to different gene expression profiles, including those that control the presence of the 2Fe-2S ferredoxin protein. Over time, organisms adapt and evolve, leading to the acquisition or loss of specific traits such as 2Fe-2S ferredoxin. This adaptation is often driven by the selective pressures of the environment and the functional needs of the organism. Keeping abreast of the latest research will provide a more comprehensive understanding of the intricate mechanisms governing the presence and functions of 2Fe-2S ferredoxin protein in different contexts.

The deficiency or excess of Fe in the human body can be involved in numerous human pathologies [107], so iron homeostasis is essential for human health. To achieve iron homeostasis, the body maintains a constant balance between iron absorption, transport, storage and distribution. Various mechanisms have evolved to control Fe homeostasis – metal chelators, Fe transporters, iron reductases, H + -ATPases and Fe chelate complexes [108,109]. In acute and chronic inflammation, under the infection, the systemic availability of iron decreases (hypoferremia). Lipopolysaccharide (LPS, endotoxin) induces a strong increase in the plasma levels of cytokines such as IL-6 and tumour necrosis factor (TNF), followed by an increase in hepcidin levels and, finally, a decrease in serum iron levels [110].

The acquisition of iron by bacteria involves various mechanisms. Bacteria acquire iron by using siderophores, by absorbing ferrous iron (after reducing ferric iron) and by using host iron compounds such as heme and transferrin. Another mechanism for ensuring access to Fe is ferroptosis. Ferroptosis is a form of cell death characterized by the iron-dependent accumulation of lipid peroxides that damage the plasma membrane [111]. Increased iron export and import have been shown to inhibit or induce ferroptosis. Carbonic anhydrases are crucial in ferroptosis, regulating iron solubility and iron transporter activities [112]. They also function in lipogenesis with protective effects on lipid peroxidation. In the context of sepsis, this mechanism could be important for the uptake of Fe by bacteria. Ferroptosis promotes infection by removing excess intracellular iron, providing the iron needed for bacterial growth. These bacteria, in turn, provide fatty acids and reactive oxygen species (ROS) needed for lipid peroxidation, leading to the exacerbation of infections, sepsis and multi-organ failure. The consequence of ferroptosis and the destructive activity of bacteria is dead cells, further recognized by immune cells. They trigger several inflammatory reactions and specific immune responses. The immune cells themselves – macrophages, T and B cells – undergo ferroptosis, which leads to dysfunction of the patient’s immune system [85]. The minimal number of study participants required to achieve power of 80% was calculated. Our study population of 64 patients with urosepsis and 85 with UTI was sufficient to demonstrate differences of 20% or more in studied biomarkers. The observed results support the hypothesis that iron uptake systems play a crucial role in the pathogenesis of urosepsis. While a larger sample size would provide even greater confidence in the findings, the strict inclusion and exclusion criteria used in patient selection helped to reduce variability and strengthen the conclusions. Future studies with expanded cohorts may further validate these findings and refine our understanding of the molecular mechanisms underlying UTI progression to urosepsis. We believe that our research provides valuable insights into the role of iron metabolism in urosepsis and highlights potential targets for diagnostic and therapeutic interventions.

## Conclusion

During a UTI, bacteria can ascend the ureter to the renal parenchyma and cross the renal tubular epithelium and capillary endothelium into the bloodstream, often causing sepsis. Little is known about the pathogenesis of urosepsis in uncomplicated and community-acquired UTI patients. Host pro-inflammatory factors and pathogen virulence in upper urinary tract infections caused by *E. coli* may lead to urosepsis. The iron uptake system of bacteria is crucial for their adaptation to the environment. The bacterial iron uptake system may play a role in bacterial survival in the patient’s urine and blood. Bacterial siderophores interact with the host to modulate cellular iron homeostasis and host immunity. Iron availability is generally reduced with inflammation, which favors expression of siderophore systems by pathogenic bacteria. This process provides an ideal habitat for bacterial modulation of the host immune response.

**Fig 6:**
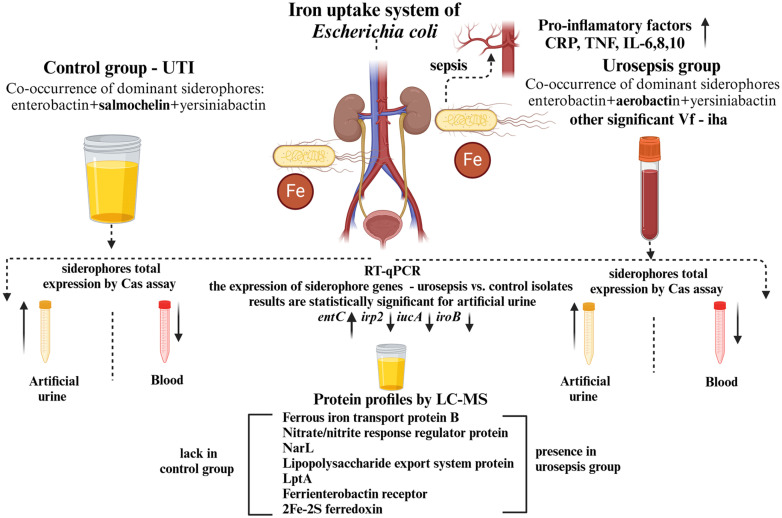
Summary results for virulence factors, pro-inflammatory factors and protein profiles for urosepsis group and control group (patients with UTI only). Figure is created in BioRender (Licence RZ26QPUF3K).

Determining the degree of bacterial virulence based on the profile of genes related to iron uptake and detecting bacterial proteins in patients’ urine, together with human markers, may provide a type of marker to assess the risk of sepsis. The link between the bacterial iron uptake system and iron homeostasis in the human body and its association with the pro-inflammatory system may be crucial in explaining the pathomechanism of urosepsis in the future. A summary of the research results is compiled on Fig 6.

## Supporting information

S1 File S1 Fig The research flow chart for *E. coli* strains isolated from the control group (with UTI) and UTI-associated urosepsis group. S2 Fig Scheme of research strain collection from patients with suspicion of urosepsis. Figure is created in BioRender (Licence RZ26QPUF3K). S3 Fig Representative genotyping results of *E. coli* using the PCR-MP fingerprinting method. Isolates were obtained from the blood and urine of patients with sepsis. Isolates from urine with the same DNA pattern as isolates from blood suggested translocation from the urinary system to the bloodstream and confirm urosepsis. Legend: U – urine; B – blood. S4 Fig PCR-MP electrophoresis patterns of *E. coli* strains isolated from individual patients with urosepsis to exclude nosocomial infection. The patterns obtained from the electrophoregram were converted and analysed using CLIQS 1D image analysis software, version 1.5.169. Cluster analysis to determine DNA relatedness was calculated using the unweighted pair group method with mathematical averaging (UPGMA). A cut-off of 86% (Po) was used for genotypes unrelated to the definition of nosocomial infection. On the left of the dendrogramme are the genes encoding the individual siderophores, for each strain separately. S5 Fig Graphical comparison of co-occurrence of genes encoding 4 types of siderophores between the urosepsis and control groups. % of strains are presented. Ent – enterobactin; Sal – salmochelin; Aer – aerobactin; Ybt – yersiniabactin. UTI – urinary tract infection (control group). S1 Table. Siderophores, their genes, and sequence primers used in PCR. S2 Table. Primer sequences and the optimized annealing temperature for primers in real-time PCR. S3 Table. The optical density (O.D.) of bacterial cultures for proteomic profiles. S4 Table. Co-occurrence of siderophore system encoding genes among *E. coli* isolates. S5 Table. Comparison of the activity of siderophore biosynthesis genes for urosepsis and control groups based on the RT qPCR method taking into account different growth conditions. S6 Table. The control group of patients and their urine isolates and the group of patients and their blood isolates were taken to study of total expression of siderophore biosynthesis genes (CAS assay). S7 Table. Variation of siderophore activities based on the CAS assay for the control group and the urosepsis group depending on the cultivation conditions. S8 Table. Proteins that distinguish the strains of *E. coli* that are responsible for urosepsis in comparison to the control group. S1 Text. Real-time PCR experiment. S2 Text. Artificial urine, M9 and M9 with blood preparation protocols. S3 Text. The artificial urine and M9 medium for the proteomic study. S4 Text. Study of relative expression of siderophore biosynthesis genes regarding the growth phase for urosepsis isolates. S5 Text. Comparison of changes in gene expression levels in *E. coli* isolates from patients’ urine and blood – selection of growth phase. (PDF)

S2 File S4_raw_images. (PDF)

S3 File S1 Data. Genetic _data set. (XLSX)

## Abbreviations

UTI: Urinary Tract Infection - Infection in any part of the urinary system
CAS assay: Chrome Azurol S assay - Used to detect and quantify siderophores
RT-qPCR: Reverse Transcription quantitative Polymerase Chain Reaction - Measures gene expression
IL-6, IL-8, IL-10, TNF: Interleukins (IL) and Tumor Necrosis Factor (TNF) - Cytokines involved in inflammation and immune responses
CRP: C-reactive Protein - Produced in response to inflammation
NGAL: (Lcn2; Scn), Neutrophil Gelatinase-Associated Lipocalin - Binds to bacterial siderophores (Lipocalin-2; Siderocalin)
HGT: Horizontal Gene Transfer - Transfer of genetic material between organisms
Vfs: Virulence Factors - Genes or proteins that enable bacteria to cause disease
UPEC: Uropathogenic *Escherichia coli - Escherichia coli* strains adapted to cause UTIs
PAI: Pathogenicity Island - Genetic elements contributing to pathogenicity in bacteria
Ybt: Yersiniabactin siderophore
Ent: Enterobactin siderophore
Sal: Salmochelin siderophore
