# Supplementary material for: Iron uptake by Escherichia coli in urinary tract infections and urosepsis
Source: PLoS One. 2025 Jun 26;20(6):e0326251. doi: 10.1371/journal.pone.0326251 (PMC12200846; doi:10.1371/journal.pone.0326251)
Supplement: S1 File — (PDF) [file pone.0326251.s001.pdf]

SUPPORTING INFORMATION FILE 1

Iron uptake by *Escherichia coli* in urinary tract infections and urosepsis

Beata Krawczyk[1,2]*, Paweł Wityk[1,2], Agnieszka Laskowska[1], Anna Stanisławska-Sachadyn[1], Joanna Raczak-Gutknecht[2], Małgorzata Waszczuk-Jankowska[2], Magdalena Burzyńska[1] , Marek Bronk[3], Tomasz Majchrzak[4], Michał J. Markuszewski[2]

[1] Department of Biotechnology and Microbiology, Faculty of Chemistry, Gdańsk University of Technology, Gdańsk, Poland

[2] Department of Biopharmaceutics and Pharmacodynamics, Medical University of Gdańsk, Gdańsk, Poland

[3] Clinical Microbiology Laboratory, Medical University of Gdańsk, Gdańsk, Poland.

[4]Department of Analytical Chemistry, Faculty of Chemistry, Gdańsk University of Technology, Gdańsk, Poland

*** Correspondence:**

email: beata.krawczyk@pg.edu.pl

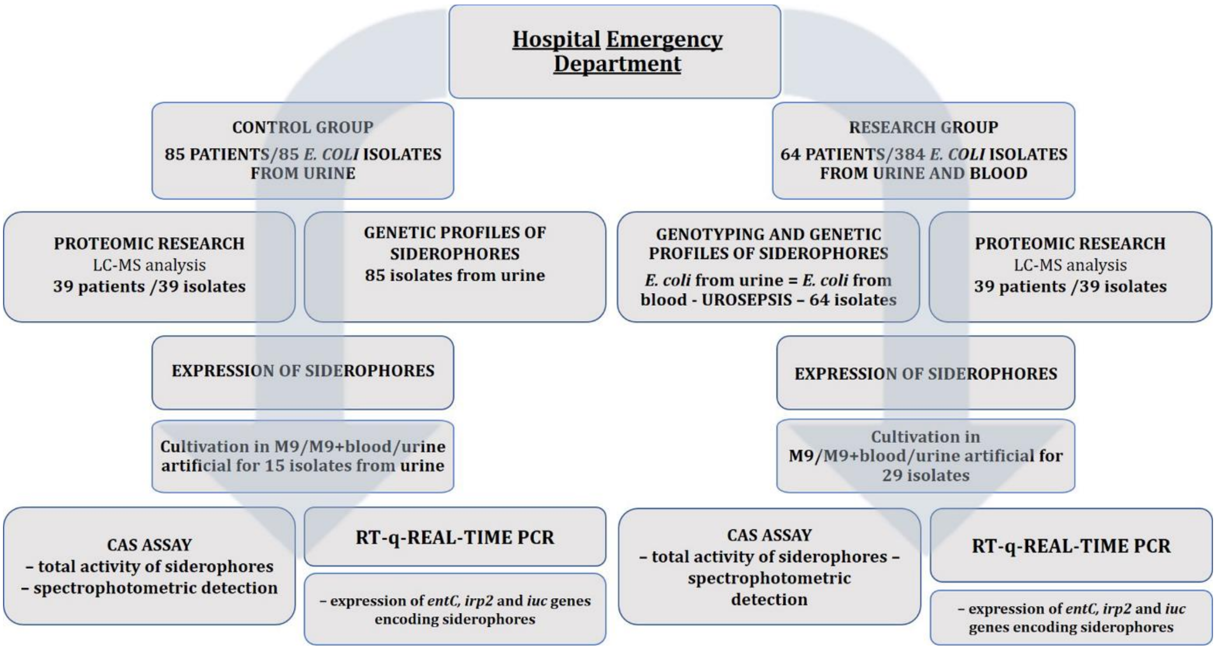

S1 Fig. The research flow chart for *E. coli* strains isolated from the control group (with UTI) and UTI-associated urosepsis group.

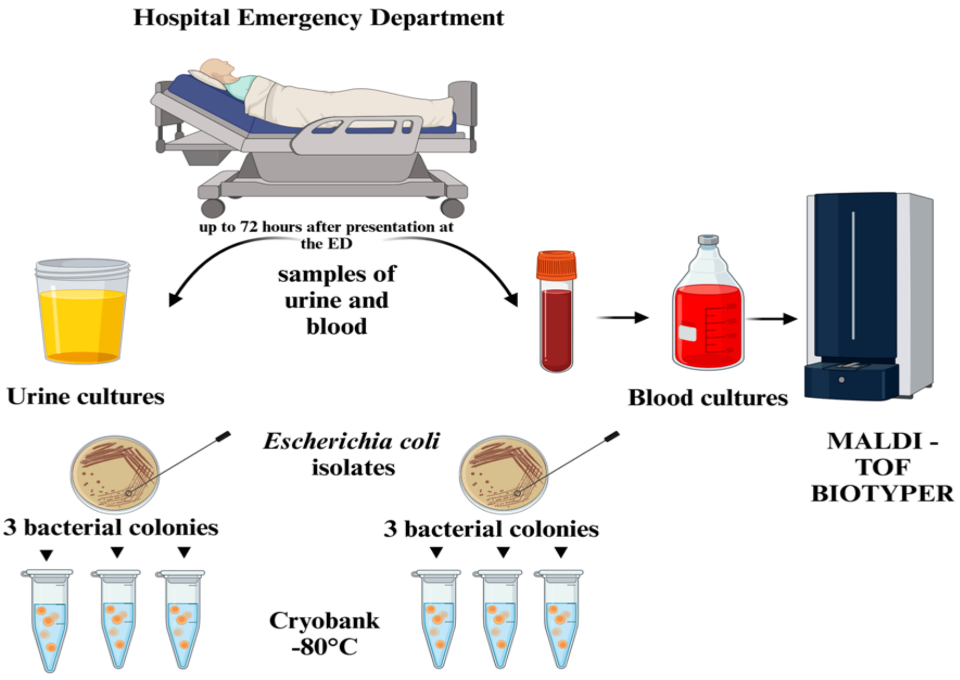

S2 Fig. Scheme of research strain collection from patients with suspicion of urosepsis. Figure is created in BioRender (Licence RZ26QPUF3K).

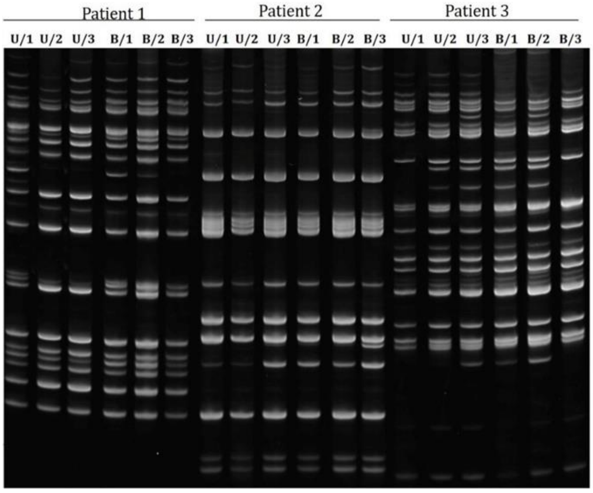

S3 Fig. Representative genotyping results of *E. coli* using the PCR-MP fingerprinting method. Isolates were obtained from the blood and urine of patients with sepsis. Isolates from urine with the same DNA pattern as isolates from blood suggested translocation from the urinary system to the bloodstream and confirm urosepsis. Legend: U - urine; B - blood.

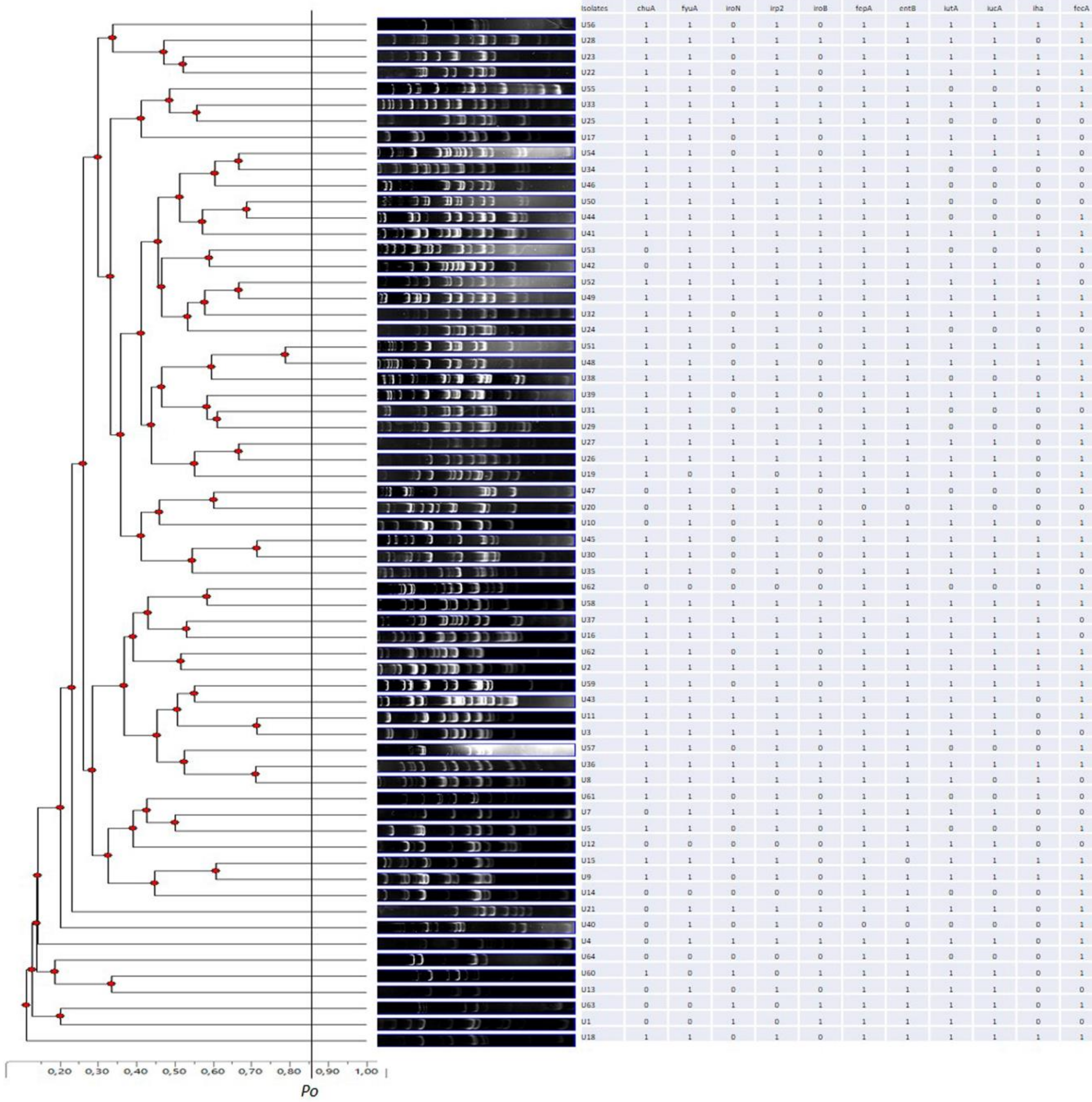

Isolates	chuA	fyuA	iroN	irp2	iroB	fepA	entB	iutA	iucA	iha	fecA
U56	1	1	0	1	0	1	1	1	1	1	1
U28	1	1	1	1	1	1	1	1	1	0	1
U23	1	1	0	1	0	1	1	1	1	1	1
U22	1	1	0	1	0	1	1	1	1	1	1
U55	1	1	0	1	0	1	1	0	0	0	1
U33	1	1	1	1	1	1	1	1	1	1	1
U25	1	1	1	1	1	1	1	0	0	0	0
U17	1	1	0	1	0	1	1	1	1	1	0
U54	1	1	0	1	0	1	1	1	1	1	0
U34	1	1	1	1	1	1	1	0	0	0	0
U46	1	1	1	1	1	1	1	0	0	0	0
U50	1	1	1	1	1	1	1	0	0	0	0
U44	1	1	1	1	1	1	1	0	0	0	1
U41	1	1	1	1	1	1	1	1	1	1	1
U53	0	1	1	1	1	1	1	0	0	0	1
U42	0	1	1	1	1	1	1	1	1	0	0
U52	1	1	1	1	1	1	1	1	1	1	0
U49	1	1	1	1	1	1	1	1	1	1	1
U32	1	1	0	1	0	1	1	1	1	1	1
U24	1	1	1	1	1	1	1	0	0	0	0
U51	1	1	0	1	0	1	1	1	1	1	1
U48	1	1	0	1	0	1	1	1	1	1	1
U38	1	1	1	1	1	1	1	0	0	0	1
U39	1	1	0	1	0	1	1	1	1	1	1
U31	1	1	0	1	0	1	1	0	0	0	0
U29	1	1	1	1	1	1	1	0	0	0	1
U27	1	1	1	1	1	1	1	1	1	0	1
U26	1	1	1	1	1	1	1	1	1	0	1
U19	1	0	1	0	1	1	1	1	1	0	1
U47	0	1	0	1	0	1	1	0	0	0	1
U20	0	1	1	1	1	0	0	1	0	0	0
U10	0	1	0	1	0	1	1	1	1	0	1
U45	1	1	0	1	0	1	1	1	1	1	1
U30	1	1	0	1	0	1	1	1	1	1	1
U35	1	1	0	1	0	1	1	1	1	1	0
U62	0	0	0	0	0	1	1	0	0	0	1
U58	1	1	1	1	1	1	1	1	1	1	1
U37	1	1	1	1	1	1	1	1	1	1	0
U16	1	1	1	1	1	1	1	1	1	1	0
U62	1	1	0	1	0	1	1	1	1	1	1
U2	1	1	1	1	1	1	1	1	1	1	1
U59	1	1	0	1	0	1	1	1	1	1	1
U43	1	1	1	1	1	1	1	1	1	0	1
U11	1	1	1	1	1	1	1	1	1	0	1
U3	1	1	1	1	1	1	1	1	1	0	0
U57	1	1	0	1	0	1	1	0	0	0	1
U36	1	1	1	1	1	1	1	1	1	1	1
U8	1	1	1	1	1	1	1	1	0	1	0
U61	1	1	0	1	0	1	1	0	0	1	0
U7	0	1	1	1	1	1	1	1	1	0	0
U5	1	1	0	1	0	1	1	0	0	0	1
U12	0	0	0	0	0	1	1	1	1	0	0
U15	1	1	1	1	0	1	0	1	1	1	1
U9	1	1	0	1	0	1	1	1	1	1	1
U14	0	0	0	0	0	1	1	0	0	0	1
U21	0	1	1	1	1	1	1	1	1	0	1
U40	0	1	0	1	0	0	0	0	0	0	1
U4	0	1	1	1	1	1	1	1	1	0	1
U64	0	0	0	0	0	1	1	0	0	0	1
U60	1	0	1	0	1	1	1	1	1	0	1
U13	0	1	0	1	0	1	1	1	1	0	0
U63	0	0	1	0	1	1	1	1	1	0	1
U1	0	0	1	0	1	1	1	1	1	0	0
U18	1	1	0	1	0	1	1	1	1	1	1

S4 Fig. PCR-MP electrophoresis patterns of *E. coli* strains isolated from individual patients with urosepsis to exclude nosocomial infection. The patterns obtained from the electrophoregram were converted and analysed using CLIQS 1D image analysis software, version 1.5.169. Cluster analysis to determine DNA relatedness was calculated using the unweighted pair group method with mathematical averaging (UPGMA). A cut-off of 86% (Po) was used for genotypes unrelated to the definition of nosocomial infection. On the left of the dendrogramme are the genes encoding the individual siderophores, for each strain separately. Legend: U - urosepsis isolates; *chuA*, *fyuA*, *iroN*, *irp-2*, *iroB*, *fepA*, *entB*, *iutA*, *iucA*, *iha*, *fecA* - genes related to iron uptake for *E. coli* strain with assigned genotype; '+' indicates presence of gene; '-' indicates absence of gene.

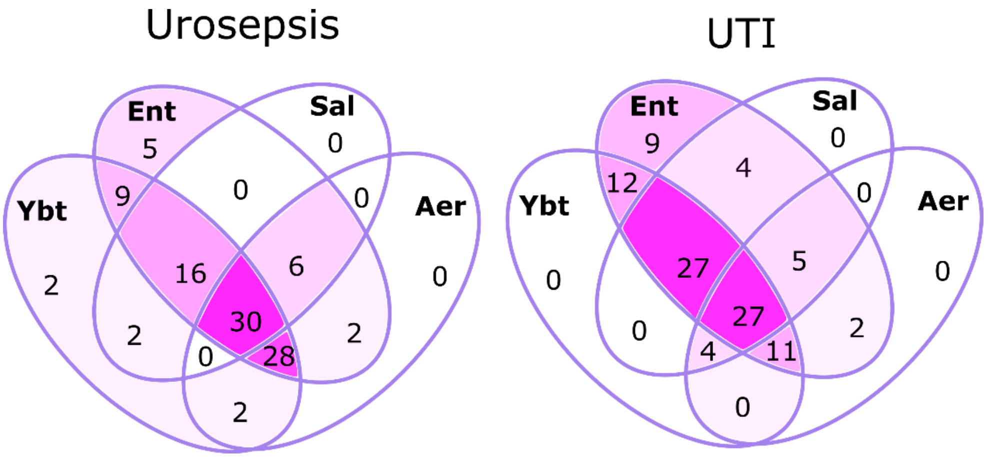

S5 Fig. Graphical comparison of co-occurrence of genes encoding 4 types of siderophores between the urosepsis and control groups. % of strains are presented. Ent – enterobactin; Sal – salmochelin; Aer – aerobactin; Ybt – yersiniabactin. UTI – urinary tract infection (control group).

S1 Table. Enzymes of siderophores metabolism, their genes, and primers' sequences used in PCR.

**Siderophores**				
**Gene**	**Function**	**Primer sequence (5'->3')**	**PCR Product (bp)**	**Reference**
entB	Synthesis enterobactin	F: CGACTACTGCAAACAGCAC<br>R: TTCAGCGACA TCAAA TGCTC	382	(Searle *et al*., 2015) (Pilarczyk-Zurek *et al*., 2016)
fepA	Ferric enterobactin receptor	F: AGCTGACTGACAGCACCATCG<br>R: GGGATGATCGACAAACGGTCG	554	(Searle *et al*., 2015)
iroB	Synthesis salmochelin	F: TATACCGGTCGTGATGCAAA<br>R: ATACTCGGCGGTGTTACGTC	150	
iroN	Ferric salmochelin receptor	F: CTTCCTCTACCAGCCTGACG<br>R: GCTCCGAAGTGATCATCCAT	648	
iucA	Synthesis aerobactin	F: ATAAGGGAAATAGCGCAGCA<br>R: TTACGGCTGAAGCGGATTAC	212	
iutA	Ferric aerobactin receptor	F: GGCTGGACATCATGGGAACTGG<br>R: CGTCGGGAACGGGTAGAATCG	302	
irp-2	Synthesis yersiniabactin	F: CTGGTGA TGGTGA TGGAAAA<br>R: CCATCGCGATAAATTGTCCT	247	
fyuA	Ferric yersiniabactin receptor	F: TGATTAACCCCGCGACGGGAA<br>R: CGCAGTAGGCACGATGTTGTA	787	
**Other factors involved in receiving and accessing iron**				
hlyA	Toxin – blood hemolysis	F: AACAACGATCCGCACTGTTCTGGCT<br>R: ACCATATAAGCGGTCATTCCCATCA	1177	(Adamus-Bialek *et al*., 2009)
chuA	Hemin receptor	F: ATGGTACCGGACGAACCAAC<br>R: TGCCGCCAGTACCAAAGACA	288	(Clermont *et al*., 2013)
iha	Bifunctional enterobactin receptor/adhesin protein	F: CTGGCGGAGGCTCTGAGATCA<br>R:TCCTTAAGCTCCCGCGGCTGA	827	(Krawczyk *et al*., 2015)
fecA	Outer membrane receptor - binding of ferric citrate	F:AGGTTAATATCGCACCGGGATCG<br>R: ATG GCA TCC ATG TTG CCG AGC	565	(Pilarczyk-Zurek *et al*., 2016)

S2 Table. Primer sequences, products of PCR and the optimized annealing temperature for primers in real-time PCR.

Siderophores/reference gene	Genes	Sequence primers (5'->3')	Product size [bp]	References	T_a (Opt)* [ºC]
enterobactin	*entC*	F: CGAGCGTTTTAGCTCCATTC	143	(Searle *et al.*, 2015)	52
		R: CCTCTTTCATCGCCTGAGTC			
salmochelin	*iroB*	F: TATACCGGTCGTGATGCAAA	150		50
		R:ATACTCGGCGGTGTTACGTC			
yersiniabactin	*irp2*	F: TAAAACTGAAGCCGGGTCAC	122		51
		R: CCGTTGTGTCACCAGAAATG			
aerobactin	*iucA*	F: CTGCCGGTCGGATTTATTTA	138		50
		R: ATAAGGGAAATAGCGCAGCA			
factor RpoB	*rpoB*	F: GTGGTGAAACCGCATCTTTT	138		51
		R: CGATGTACTCAACCGGGACT			

*Ta (Opt) – optimal the annealing temperature for primers.

References to S1 and S2 Table:

Adamus-Bialek W, Wojtasik A, Majchrzak M, Sosnowski M, Parniewski P. (CGG)4-based PCR as a novel tool for discrimination of uropathogenic *Escherichia coli* strains: comparison with enterobacterial repetitive intergenic consensus-PCR. *J Clin Microbiol*. 2009 Dec;47(12):3937-44. doi: 10.1128/JCM.01036-09. Epub 2009 Oct 21. PMID: 19846645; PMCID: PMC2786682

Clermont O, Christenson JK, Denamur E, Gordon DM. The Clermont Escherichia coli phylo-typing method revisited: improvement of specificity and detection of new phylo-groups. *Environ Microbiol Rep.* 2013 Feb;5(1):58-65. doi: 10.1111/1758-2229.12019. Epub 2012 Dec 24. PMID: 23757131.

Krawczyk B, Śledzińska A, Szemiako K, Samet A, Nowicki B, Kur J. Characterisation of Escherichia coli isolates from the blood of haematological adult patients with bacteraemia: translocation from gut to blood requires the cooperation of multiple virulence factors. *Eur J Clin Microbiol Infect Dis.* 2015 Jun;34(6):1135-43. doi: 10.1007/s10096-015-2331-z. Epub 2015 Feb 6. PMID: 25655758; PMCID: PMC4426128.

Pilarczyk-Zurek M, Strus M, Adamski P, Heczko PB. The dual role of Escherichia coli in the course of ulcerative colitis. BMC Gastroenterol. 2016 Oct 10;16(1):128. doi: 10.1186/s12876-016-0540-2. PMID: 27724868; PMCID: PMC5057264.

Searle LJ, Méric G, Porcelli I, Sheppard SK, Lucchini S. Variation in siderophore biosynthetic gene distribution and production across environmental and faecal populations of Escherichia coli. PLoS One. 2015 Mar 10;10(3):e0117906. doi: 10.1371/journal.pone.0117906. PMID: 25756870; PMCID: PMC4355413.

S3 Table. The optical density (O.D.) of bacterial cultures for proteomic profiles.

CONTROL GRUP (UTI)				UROSEPSIS			
Name	OD_{600}	Name	OD_{600}	Name	OD_{600}	Name	OD_{600}
C1	0.410	C21	0.629	U1	0.601	U21	0.515
C2	0.488	C22	0.500	U2	0.594	U22	0.553
C3	0.335	C23	0.410	U3	0.537	U23	0.447
C4	0.449	C24	0.405	U4	0.527	U24	0.513
C5	0.492	C25	0.470	U5	0.509	U25	0.603
C6	0.425	C26	0.370	U6	0.529	U26	0.405
C7	0.586	C27	0.420	U7	0.425	U27	0.517
C8	0.434	C28	0.620	U8	0.631	U28	0.500
C9	0.505	C29	0.665	U9	0.623	U29	0.488
C10	0.534	C30	0.455	U10	0.592	U30	0.642
C11	0.478	C31	0.551	U11	0.551	U31	0.534
C12	0.403	C32	0.668	U12	0.494	U32	0.488
C13	0.359	C33	0.609	U13	0.531	U33	0.559
C14	0.369	C34	0.623	U14	0.476	U34	0.511
C15	0.442	C35	0.690	U15	0.655	U35	0.504
C16	0.514	C36	0.698	U16	0.595	U36	0.604
C17	0.439	C37	0.598	U17	0.483	U37	0.400
C18	0.384	C38	0.608	U18	0.539		
C19	0.485	C39	0.637	U19	0.536		
C20	0.554			U20	0.535		

S4 Table. Co-occurrence of siderophore system encoding genes among *E. coli* isolates.

	**Isolates from UTI patients (control group) n=85**			**Isolates from urosepsis patients (research group) n=64**			***P-value***
No system of siderophores	Co-occurrence of siderophores	No. of strains [n]	% of strains	Co-occurrence of siderophores	No. of strains [n]	% of strains	
1	Ent	8	9	Ent	3	5	0.4068
	Sal	0	0	Sal	0	0	1.
	Ybt	0	0	Ybt	1	1.5	0.4295
	Aer	0	0	Aer	0	0	1.
2	Ent+Ybt	10	12	Ent+Ybt	6	9	0.791
	Ent+Sal	3	4	Ent+Sal	0	0	0.1212
	Ent+Aer	2	2	Ent+Aer	1	2	1.
	Ybt+Aer	0	0	Ybt+Aer	1	1.5	0.4295
	Ybt+Sal	0	0	Ybt+Sal	1	1.5	0.4295
	Sal+Aer	0	0	Sal+Aer	0	0	1.
3	**Ent+Sal+Ybt**	**23**	**27**	**Ent+Sal+Ybt**	**10**	**16**	**0.0408**
	Ent+Sal+Aer	4	5	Ent+Sal+Aer	4	6	1.
	Sal+Aer+Ybt	3	3.6	Sal+Aer+Ybt	0	0	0.2599
	**Ent+Aer+Ybt**	**9**	**11**	**Ent+Aer+Ybt**	**18**	**28**	**0.0039**
4	Ent+Sal+Ybt+Aer	23	27	Ent+Sal+Ybt+Aer	19	30	0.7542
	Total	**85**		Total	**64**		

Legend: *Included only biosynthesis genes; Fisher Exact Test; Significance Level (P): ≤0.05

S5 Table. Comparison of the activity of siderophore biosynthesis genes for urosepsis and control groups based on the RT qPCR method taking into account different growth conditions.

Gene	Group	M9			M9 with blood			Artificial urine		
		Mean of expression gene	SEM	P*	Mean of expression gene	SEM	P*	Mean of expression gene	SEM	P*
entC	Urosepsis	0.982	0.269	0.104	0.685	0.077	0.431	0.899	0.123	0.029
	Control	0.855	0.131		0.658	0.110		0.519	0.106	
irp-2	Urosepsis	0.090	0.034	0.869	0.076	0.016	0.097	0.019	0.008	0.007
	Control	0.018	0.009		0.366	0.154		0.048	0.013	
iucA	Urosepsis	2.560	0.789	0.979	1.945	0.349	0.633	4.359	1.789	0.030
	Control	1.453	0.306		1.812	1.341		9.867	1.521	
iroB	Urosepsis	0.033	0.009	0.001	3.894	1.031	0.813	0.068	0.027	0.012
	Control	1.310	0.538		4.853	2.904		0.189	0.055	

P* by Mann-Whitney U test.

S6 Table. The control group of patients and their urine isolates and the group of patients and their blood isolates were taken to study of total expression of siderophore biosynthesis genes (CAS assay).

Control group n=15					
**Number of isolates**	**Sp % M9_CAS**	**Sp % M9+blood_CAS**	**Sp % artificial urine_CAS**	**Genes of siderophores**	**number of siderophore genes**
C1	88.513	87.758	92.662	*entB*	1
C2	84.29	89.675	95.394	*entB*	1
C3	85.642	81.858	92.272	*irp2. entB*	2
C4	90.372	85.84	92.272	*irp2. entB*	2
C5	86.486	86.283	93.833	*iroB. entB*	2
C6	87.838	86.725	94.301	*iroB. entB. iucA*	3
C7	86.486	81.563	92.506	*irp2. entB.iucA*	3
C8	85.135	80.383	91.725	*iroB.entB.iucA*	3
C9	84.797	85.988	94.692	*irp2. iroB.entB*	3
C10	83.784	86.873	93.833	*irp2. iroB.entB*	3
C11	90.878	89.233	93.599	*irp2. iroB.entB.iucA*	4
C12	86.486	82.448	93.052	*irp2. iroB.entB.iucA*	4
C13	88.851	88.643	93.755	*irp2. iroB.entB.iucA*	4
C14	87.838	86.578	94.223	*irp2. iroB.entB.iucA*	4
C15	88.007	88.053	93.286	*irp2. iroB.entB.iucA*	4
**Urosepsis group n=29**					
**Number of isolates**	**Sp % M9_CAS**	**Sp % M9+blood_CAS**	**Sp % artificial urine_CAS**	**genes**	**number of siderophore genes**
U1	87.162	91.15	95.628	*entB*	1
U2	92.736	88.201	93.911	*irp2*	1
U3	91.554	86.873	95.004	*irp2 entB*	2
U4	90.878	89.233	96.721	*entB iucA*	2
U5	87.838	87.168	98.595	*irp2 iucA*	2
U6	93.243	81.858	93.755	*irp2 iroB*	2
U7	88.176	83.776	96.409	*iroB. entB iucA*	3
U8	91.554	87.021	95.394	*irp2 entB iucA*	3
U9	84.291	86.431	95.628	*irp2 iroB entB*	3
U10	90.203	91.15	93.13	*irp2 entB iucA*	3
U11	93.75	90.708	94.379	*irp2 entB iucA*	3
U12	90.372	91.445	95.16	*irp2 entB iucA*	3
U13	92.905	86.283	95.238	*irp2 entB iucA*	3
U14	87.669	87.906	97.19	*irp2 entB iucA*	3
U15	88.682	85.251	95.785	*iroB.entB iucA*	3
U16	94.595	93.068	95.16	*irp2 iroB entB*	3

U1729	92.061	89.381	94.926	*irp2 entB iucA*	3
U18	93.074	82.153	96.331	*irp2 iroB entB*	3
U19	86.993	84.513	94.379	*irp2 iroB entB*	3
U20	90.709	84.366	97.19	*irp2 iroB entB*	3
U21	88.682	93.658	96.175	*irp2 iroB ent iucA*	4
U22	90.709	84.956	94.614p2	*irp2 iroB entB iucA*	4
U23	90.372	84.956	97.19	*irp2. iroB.entB.iucA*	4
U24	89.527	82.891	94.223	*irp2 iroB entB iucA*	4
U25	91.723	95.28	95.004	*irp2 iroB entB iucA*	4
U26	89.865	87.168	97.58	*irp2 iroB.entB.iucA*	4
U27	86.655	88.791	95.238	*irp2 iroB.entB.iucA*	4
U28	93.074	81.563	89.852	*irp2 iroB.entB.iucA*	4
U29	92.061	89.086	96.721	*irp2 iroB entB iucA*	4

Legend: C1-C15 - control group with UTI without urosepsis. U1-U 29 - isolated from the blood patients with confirmed urosepsis.

S7 Table. Variation of siderophore activities based on the CAS assay for the control group and the urosepsis group depending on the cultivation conditions.

Group	M9			M9 with blood			Artificial urine			
	mean of Sp%	SEM	P*	mean of Sp%	SEM	P*	mean of Sp%	SEM	P*	P
**Urosepsis**	90.383	0.461	0	87.458	0.669	0.202	95.397	0.307	<0.0001	P^1<0.0001
**Control**	87.027	0.548		85.860	0.758		93.427	0.263		P^2<0.0001

Legend: SEM* - Standard Error of Measurement; SD** - Standard Deviation; Sp% - percentage activity of siderophores. P* by Mann-Whitney U test ; P by Friedman test (P^1 for the urosepsis group: M9 vs M9+ blood P=0.203; M9 vs artificial urine P<0.0001; M9+blood vs artificial urine P<0.0001; P^2 control group: M9 vs M9+ blood P=0.408; M9 vs artificial urine P<0.002; M9+blood vs artificial urine P<0.0001), for analyses of paired samples.

S8 Table. Proteins that distinguish the strains of *E. coli* that are responsible for urosepsis in comparison to the control group.

Protein Name	Function	Gene Name	Control group	Urosepsis group
Bacterioferritin	This protein is involved in iron storage and transport within bacteria. It helps maintain iron homeostasis and prevent the harmful effects of iron overload.	*bfr*	+	+
Ferritin-1	Ferritins are vital for iron storage in cells. They store iron in a controlled manner, preventing the formation of harmful reactive oxygen species that can damage cells.	*ftnA*	+	↥
Iron uptake system component EfeO	IrEfeO contributes to the uptake of iron in certain bacteria. It is a part of the machinery that ensures the efficient acquisition of this crucial nutrient.	*efeO*	↥	+
Lipopolysaccharide export system ATP-binding protein	This protein is integral to the export of lipopolysaccharides, which are essential components of the outer membrane of Gram-negative bacteria. Proper export is critical for maintaining the structural integrity of bacterial cells.	*lptB*	+	+
Ferrous iron transport protein B	Involved in transporting ferrous iron, this protein aids in the movement of iron across cell membranes and is crucial for maintaining iron balance.	*feoB*	-	+
Ferric uptake regulation protein	Also known as Fur, this protein acts as a regulatory factor in controlling iron uptake genes. It helps maintain iron levels within optimal ranges.	*fur*	+	+
Nitrate/nitrite response regulator protein NarL	This protein plays a role in the response to nitrate and nitrite levels in the environment. Its functions are critical for bacterial adaptation to changing conditions.	*narL*	-	+
Protein HemY	HemY is involved in heme synthesis, which is essential for various cellular processes, including oxygen transport and electron transfer.	*hemY*	+	↥
Iron-sulfur cluster assembly scaffold protein IscU	Iron-sulfur clusters are critical co-factors in various proteins. IscU is involved in assembling and transferring these clusters to other proteins.	*iscU*	+	+
Ferrienterobactin receptor	This protein is integral to the uptake of the siderophore enterobactin, which is a molecule bacteria produced to scavenge iron from their environment.	*fepA*	-	+
Fe/S biogenesis protein NfuA	Like IscU, NfuA is involved in the assembly and transfer of iron-sulfur clusters. These clusters are essential	*nfuA*	+	+

	for the function of numerous enzymes.			
Lipopolysaccharide export system protein LptA	LptA is part of the machinery responsible for exporting lipopolysaccharides to the bacterial cell surface. This process is crucial for maintaining the integrity of the outer membrane.	*lptA*	-	+
Iron-sulfur cluster insertion protein ErpA	ErpA is involved in the insertion of iron-sulfur clusters into target proteins, ensuring their proper function.	*erpA*	+	+
2Fe-2S ferredoxin	Ferredoxins are proteins that are often involved in electron transfer reactions. The presence of iron-sulfur clusters allows them to participate in a wide range of redox reactions.	*fdx*	-	+

Legend: .”–“ lack production of this protein; ↥ assign differentiation of protein level (increased level); “+” – presence production of this protein

S1 Text. Real-time PCR experiment

From each tested isolate four samples were analysed, that is, from cultures in the logarithmic growth phase and the stationary phase grown on either M9 minimal medium or artificial urine. Four dilutions of the cDNA were prepared and amplified with each pair of primers in triplicate. This allowed to calculate amplification efficiency for each amplicon. Summary of efficiency calculations for the reference gene and the tested genes are presented in Table 1.

To confirm the specificity of the obtained products, a melting curve analysis of the amplified fragments of the *entC, iroB, irp2, iucA* and *rpoB* genes was performed (the construction of the melting curve by heating on a slow ramp between 50ºC [60s, 2,2 ºC/s] and 97ºC [60s, 0,1ºC/s]).

Table 1. Results obtained from the determination of calibrator standard curves for the reference gene and the tested genes. Correlation coefficient R2 values; PCR reaction efficiency (Efficiency).

Genes	*iroB*	*entC*	*iucA*	*irp2*	*rpoB*
**Efficiency**	1,89	1,99	1,92	1,84	1,85
**R^2**	0,97	0,92	0,98	0,97	0,94

Fig 1 shows the standard curves of the analyzed sequences of the studied genes *entB, iroB, irp2* and *iucA,* obtained using a spreadsheet.

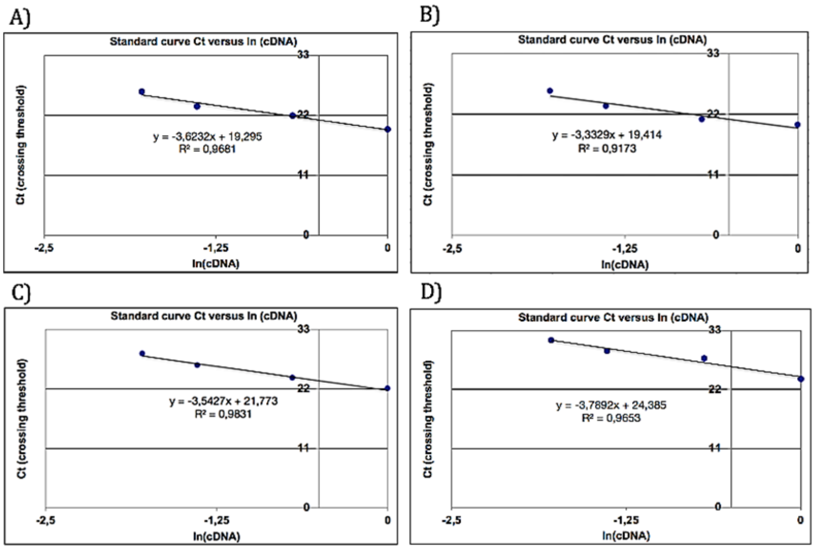

Fig 1. Standard curves for real-time PCR of the tested genes A) *iroB,* B) *entC,* C) *iucA,* D) *irp2*

S2 Text. Artificial urine, M9 and M9 with blood preparation protocols

Components for artificial urine: peptone K (BTL) (1g/L), yeast extract (0.005g/L) (BTL, Poland), lactic acid (0.1g/L) (SIGMA-ALDRICH), citric acid (0,4g/L) (SIGMA-ALDRICH), sodium bicarbonate (2.1 g/L) (SIGMA-ALDRICH), urea (10g/L) (SIGMA-ALDRICH), uric acid (0.07g/L) (SIGMA-ALDRICH), creatinine (0.8g/L) (SIGMA-ALDRICH), calcium chloride x 2 H2O (0.37g/L) (SIGMA-ALDRICH), iron II sulphate x 7 H2O (0.0012g/L) (SIGMA-ALDRICH), magnesium sulphate x 7 H2O (0,49g/l) (SIGMA-ALDRICH), sodium sulphate x 10 H2O (3.3g/L) (SIGMA-ALDRICH), di-potassium hydrogen phosphate (1.2g/L) (SIGMA-ALDRICH), potassium dihydrogen phosphate (0.95g/L) (SIGMA-ALDRICH), ammonium chloride (1.3 g/L) (SIGMA-ALDRICH), sodium chloride (5.2g/L) (STANDARD, POLAND). The pH 6,5 urine medium was achieved with hydrochloric acid. The whole was sterilized by filtration (VWR® Vacuum filtration, 150 mL, 0.2µm PES filter Unit).

The **M9 medium** (Sigma) contained disodium hydrogen phosphate 6g/L, potassium dihydrogen phosphate 3g/L, sodium chloride 0.5g/L and ammonium chloride 1g/L. 10.5g of the medium was dissolved in a liter of distilled water and the pH was adjusted to 7.4. Then, the substrate was sterilized in an autoclave at 121°C. After cooling, 2 mL of 1 M magnesium sulfate and 20 mL of 20% carbon source - glycerol were added using a syringe with a filter.

M9 supplemented with whole blood - the composition of regular M9 medium but enriched with 500 µl of human whole blood per 10 mL M9.

S3 Text. The artificial urine and M9 medium for the proteomic study

The artificial urine - preparation as in S2 Text but without peptone and yeast extract.
M9 medium - 6 g/L of disodium hydrogen phosphate (Molecular Biology Grade, Merck, Germany), 3 g/L of potassium dihydrogen phosphate (Molecular Biology Grade, Merck, Germany), 0.5 g/L of sodium chloride (Molecular Biology Grade, Merck, Germany) and 1 g/L of ammonium chloride (Molecular Biology Grade, Merck, Germany) were added and mixed until complete dissolution of the components. The final pH of the solution was adjusted to 7.4 at 25°C. The solution was then autoclaved at 121°C for 15 min. Subsequently, 2 mL of a 1 M magnesium sulfate solution (Molecular Biology Grade, Merck, Germany) and 20 mL of a 20% glycerol solution (Molecular Biology Grade, Merck, Germany) were added. Both solutions had undergone prior sterile filtration through a 0.2 μm pore diameter filter.

S4 Text. Study of relative expression of siderophore biosynthesis genes regarding the growth phase for urosepsis isolates

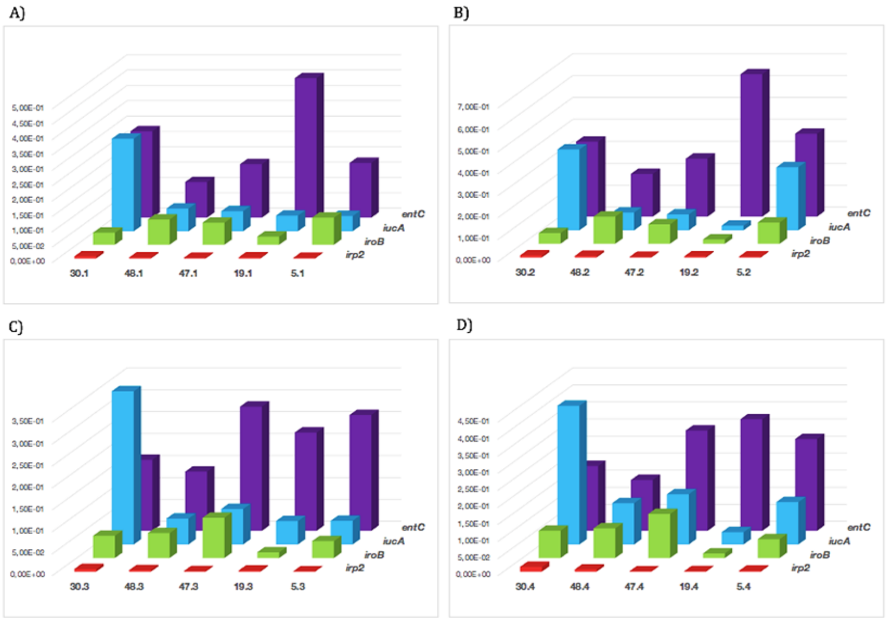

Fig 1. Relative expression level of siderophore biosynthesis genes for each tested *E. coli* strains isolated from urine of urosepsis patients. Designation of tested genes: *entC* – purple, *iucA* – blue, *iroB* – green, *irp2* – red; A) isolates grown on M9, logarythmic growth phase; B) isolates grown on M9, stationary phase, C) isolates grown on artificial urine, logarithmic growth phase, D) isolates grown on artificial urine, stationary phase. Numbers: 30, 48, 47, 19, 5 – means the isolate number from the collection (1,3 –means log phase; 2,4- means stationary phase)

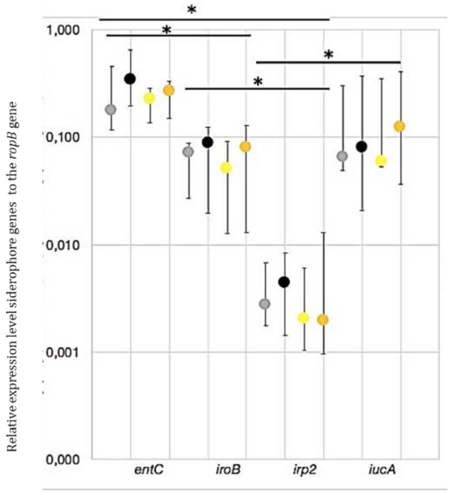

Fig 2. Relative expression level of siderophore biosynthesis genes among the tested *E. coli* strains isolated from urine of urosepsis patients. Expression level of *entC*, *iroB*, *irp2* and *iucA* genes relative to the internal reference – *rpoB* reference gene. Points on the graph represent the median, error bars represent the minimum and maximum value; *P <0.05. Grey – M9

medium, logarithmic growth phase; Black – M9 medium; stationary phase; Light yellow – arificial urine, logarithmic growth phase; Dark yellow – artificial urine, stationary phase. Statistical analysis was performed using the Mann-Whitney U test.

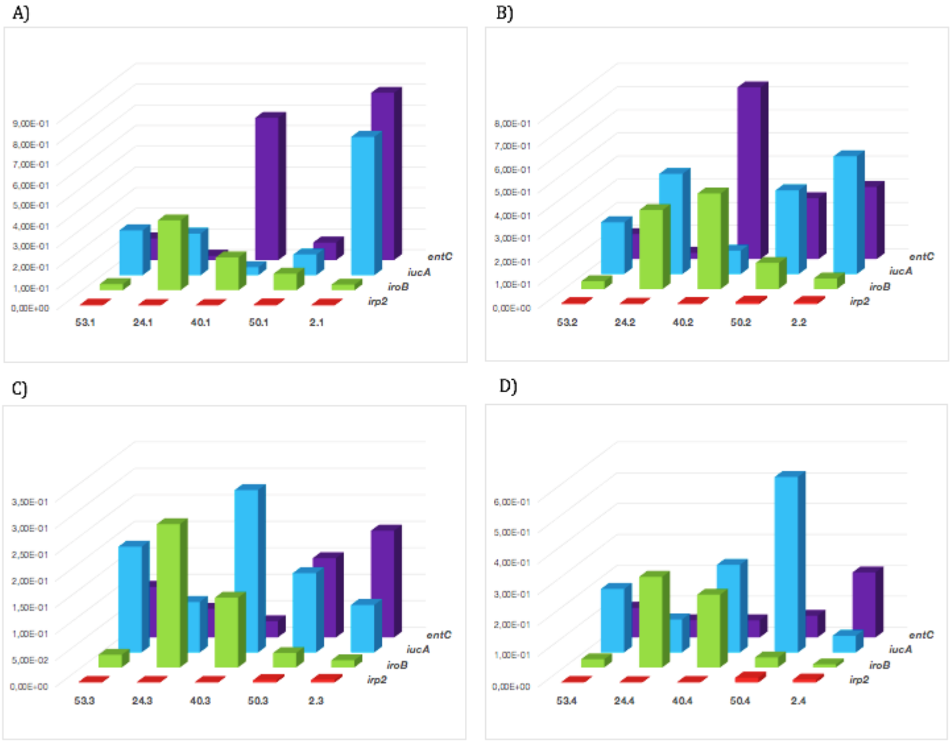

Fig 3. Relative expression level of siderophore biosynthesis genes for each tested *E. coli* strain isolated from blood. Designation of tested genes: *entC* – purple, *iucA* – blue, *iroB* – green, *irp2* – red; A) isolates grown on M9 medium, logarithmic growth phase; B) isolates grown on M9 medium, stationary phase, C) isolates grown on artificial urine, logarithmic growth phase, D) isolates grown on artificial urine, stationary phase. Numbers:53, 24, 40, 50, 2 – means the isolate number from the collection (1,3 –means log phase; 2,4- means stationary phase)

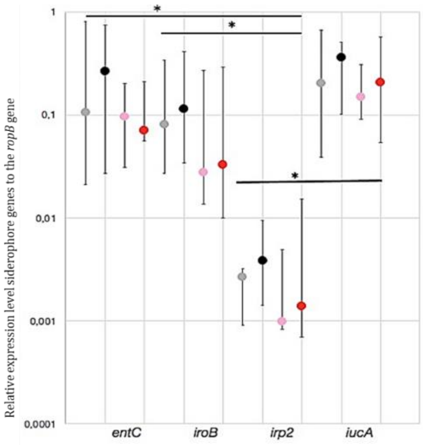

Fig 4. Relative expression level of siderophore biosynthesis genes among the tested *E. coli* strains isolated from blood of urosepsis patients. Expression level of *entC*, *iroB*, *irp2* and *iucA* genes relative to the internal reference – *rpoB* reference gene. Points on the graph represent

the median, error bars represent the minimum and maximum value; *P-value <0.05. Grey – M9 medium, logarithmic growth phase; Black – M9 medium; stationary phase; Pink – arificial urine, logarithmic growth phase; Red – artificial urine, stationary phase.

In case isolates from urine, and for all combinations of culture conditions (culture on M9 medium for the logarithmic growth phase; culture on M9 medium for the stationary phase; culture on artificial urine for the logarithmic growth phase; culture on artificial urine for the stationary phase,) a statistically significant result was recorded for the relative expression levels of the genes *entC* and *iroB, entC* and *irp2*, *iroB* and *irp2* and *irp2* and *iucA* ($P<0.05$). For isolates from blood patients according to the same cultivation condition a statistically significant result was recorded for the relative expression levels of the genes *entC* and *irp2*, *iroB* and *irp2, iucA* and *irp2* ($P<0.05$).

S5 Text. Comparison of changes in gene expression levels in *E. coli* isolates from patients' urine and blood - selection of growth phase

The averaged results of the fold changes in the expression levels of the studied genes of *E. coli* isolates collected from urine and blood of urosepsis patients are presented in Table 1. The average expression level of the genes studied is presented separately for isolates collected in logarithmic and stationary phase.

Table 1. Averaged results of relative fold changes in expression levels of siderophore biosynthesis genes. 5 isolates of *E. coli* from patients' urine and 5 isolates of *E. coli* from patients' blood after cultivation on artificial urine were studied. Isolates were examined in logarithmic and stationary phase growth.

**Artificial urine/M9 medium<br>Fold change in gene expression**				
**Genes**	***entC***	***iroB***	***irp2***	***iucA***
***E.coli* isolates from patients' urine (n=5)**				
**Logarithmic growth phase**	1,066	0,835	0,730	1,066
**Stationary phase**	0,729	1,007	0,744	1,360
***E.coli* isolates from patients' blood (n=5)**				
**Logarithmic growth phase**	1,110	0,657	1,049	2,195
**Stationary phase**	0,794	0,545	0,799	1,131

Table 2. Standard deviation (SD) for fold change in gene expression.

**Gene**	**Urine (log)/SD**	**Urine (stat)/SD**	**Blood (log)/SD**	**Blood (stat)/SD**
entC	0.32	0.14	0.51	0.41
iroB	0.33	0.41	0.34	0.41
irp2	0.51	0.46	0.4	2.1
iucA	0.21	0.19	0.23	0.11

Legend; log/stat - logarithmic growth phase/stationary growth phase

Table 3. Comparison of gene expression levels for isolates collected at logarithmic and stationary growth phases.

Gene	P_{value}	P_{value}	P_{value}	P_{value}
	Urine (n=5) (log vs stat)	Blood (n=5) (log vs stat)	Log phase (urine vs blood)	Stationary phase (urine vs blood)
entC	0.0787	0.3131	0.875	0.7511
iroB	0.4867	0.6512	0.4253	0.1127
irp2	0.9648	0.8058	0.3049	0.9569
iucA	**0.0491**	**0.00011**	4.16×10^{-5}	0.0557

Legend: log/stat logarithmic growth phase/stationary growth phase; n- number of isolates tested

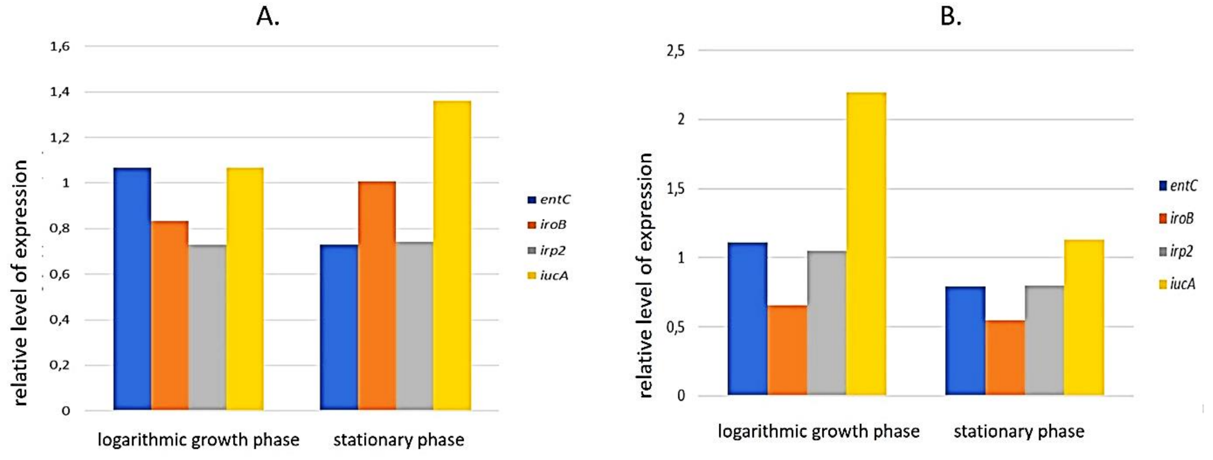

Fig 1. Changes in relative expression levels of the studied genes of *E. coli* isolates isolated from patients' urine (A) and patients' blood (B) depending on the growth phase of the microorganism.

Statistical analysis performed using the Mann-Whitney U test showed that the differences in the levels of *E. coli* gene expression between the groups of isolates in the logarithmic growth phase and the stationary phase were statistically insignificant for *entC*, *iroB*, *irp2*, both for isolates from urine and for blood. In case of *iucA* fold change in gene expression for urine and blood isolates was statistically significant ($P \leq 0.05$).
