# Supplementary material for: Iron uptake by Escherichia coli in urinary tract infections and urosepsis
Source: PLoS One. 2025 Jun 26;20(6):e0326251. doi: 10.1371/journal.pone.0326251 (PMC12200846; doi:10.1371/journal.pone.0326251)
Supplement: S2 File — (PDF) [file pone.0326251.s002.pdf]

S4_raw_images

M	1	2	3	4	5	6	7	8	9	10	11	12	13	14	15	16	17	18

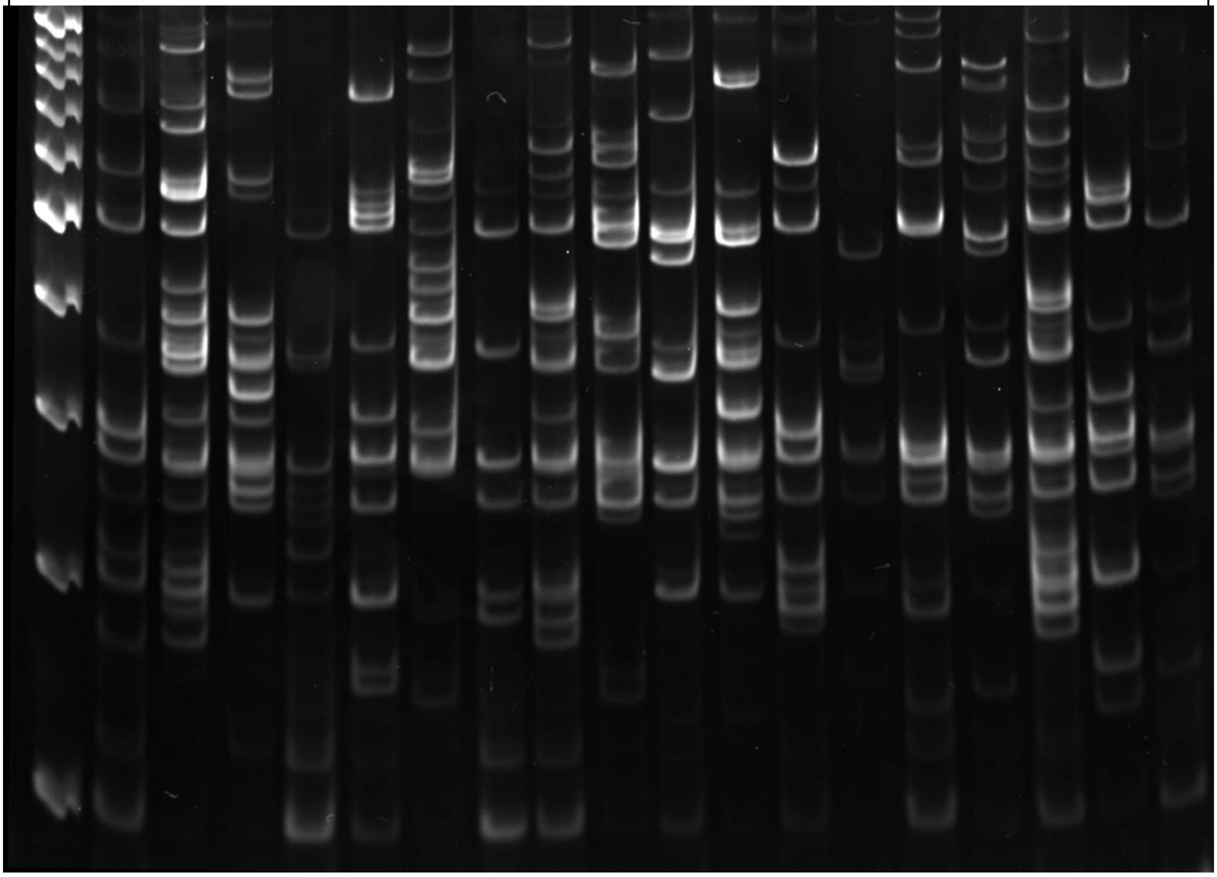

M 19 20 21 22 23 24 25 26 27 28 29 30 31 32 33 34 35 36

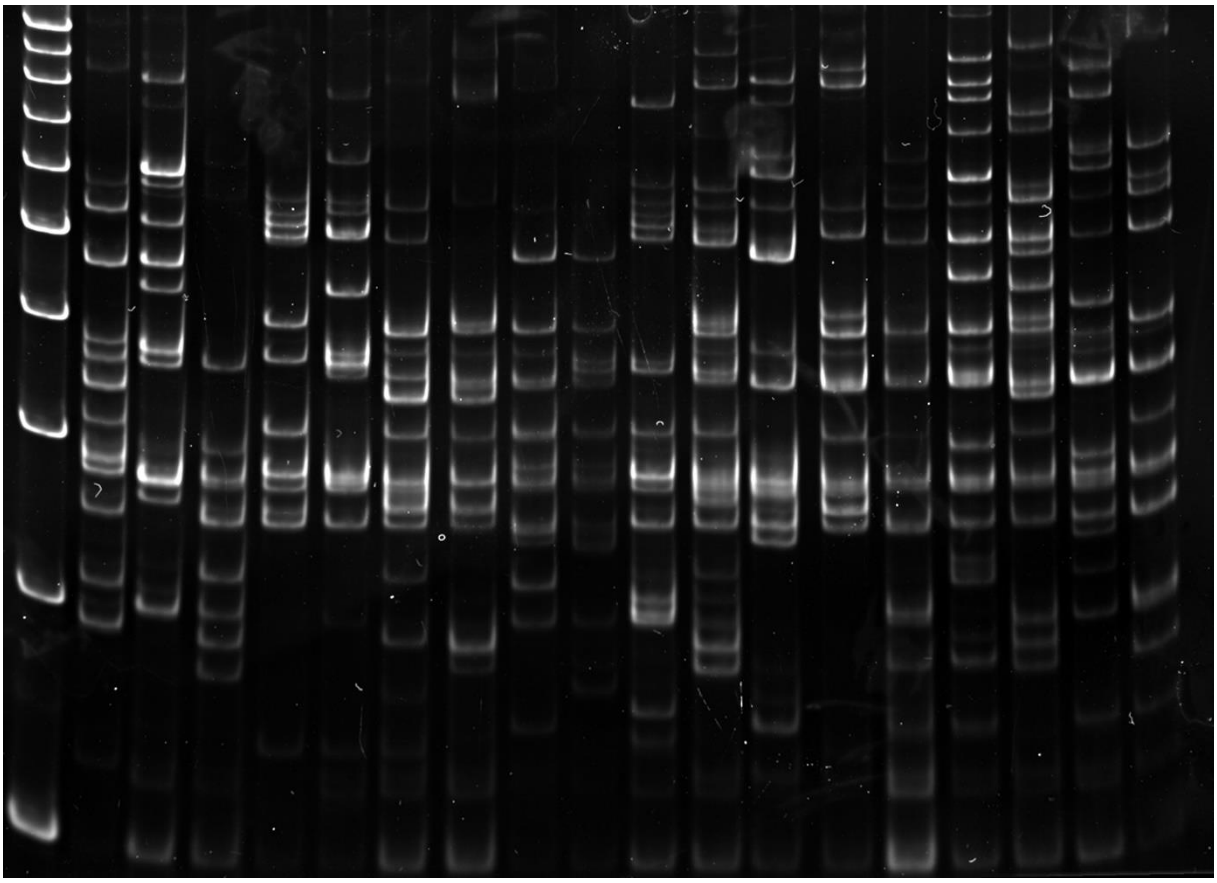

M 37 38 39 40 41 42 43 44 45 46 47 48 49 50 51 52 53 54 55

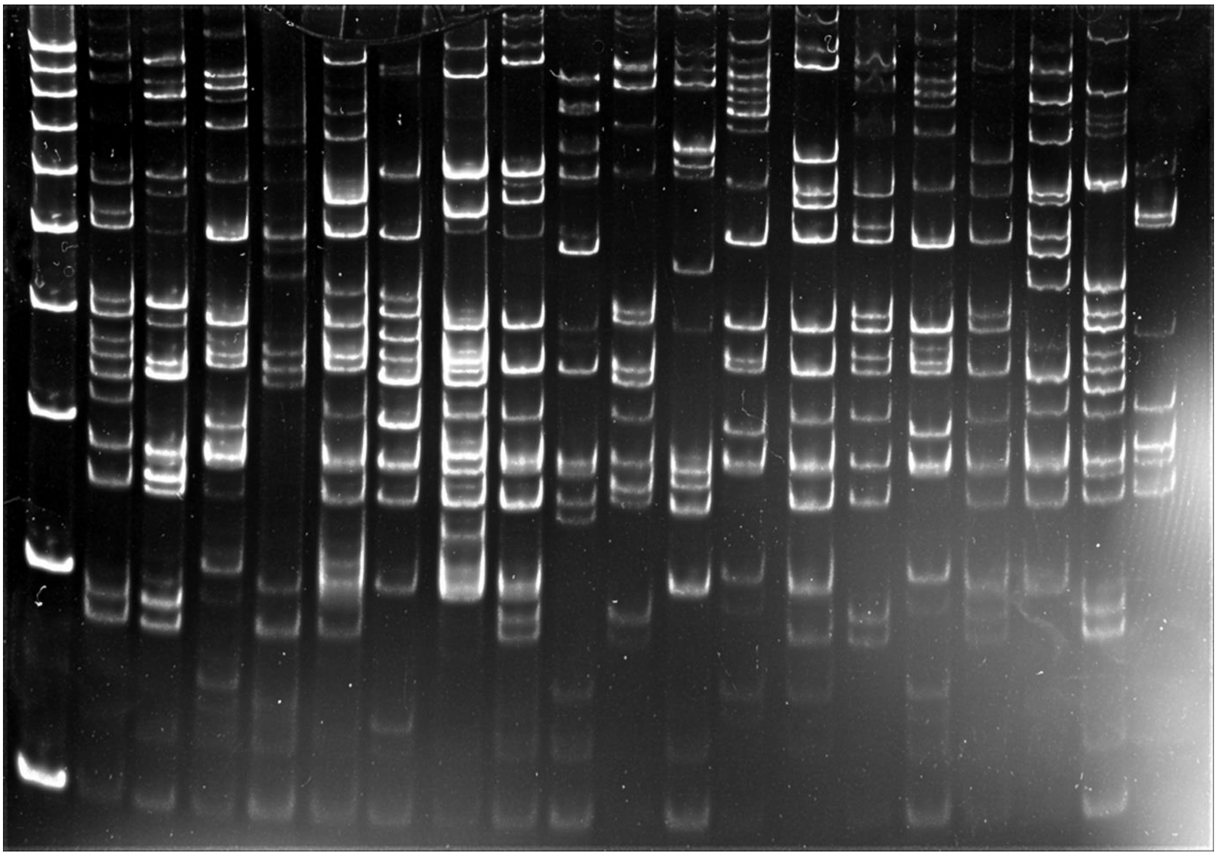

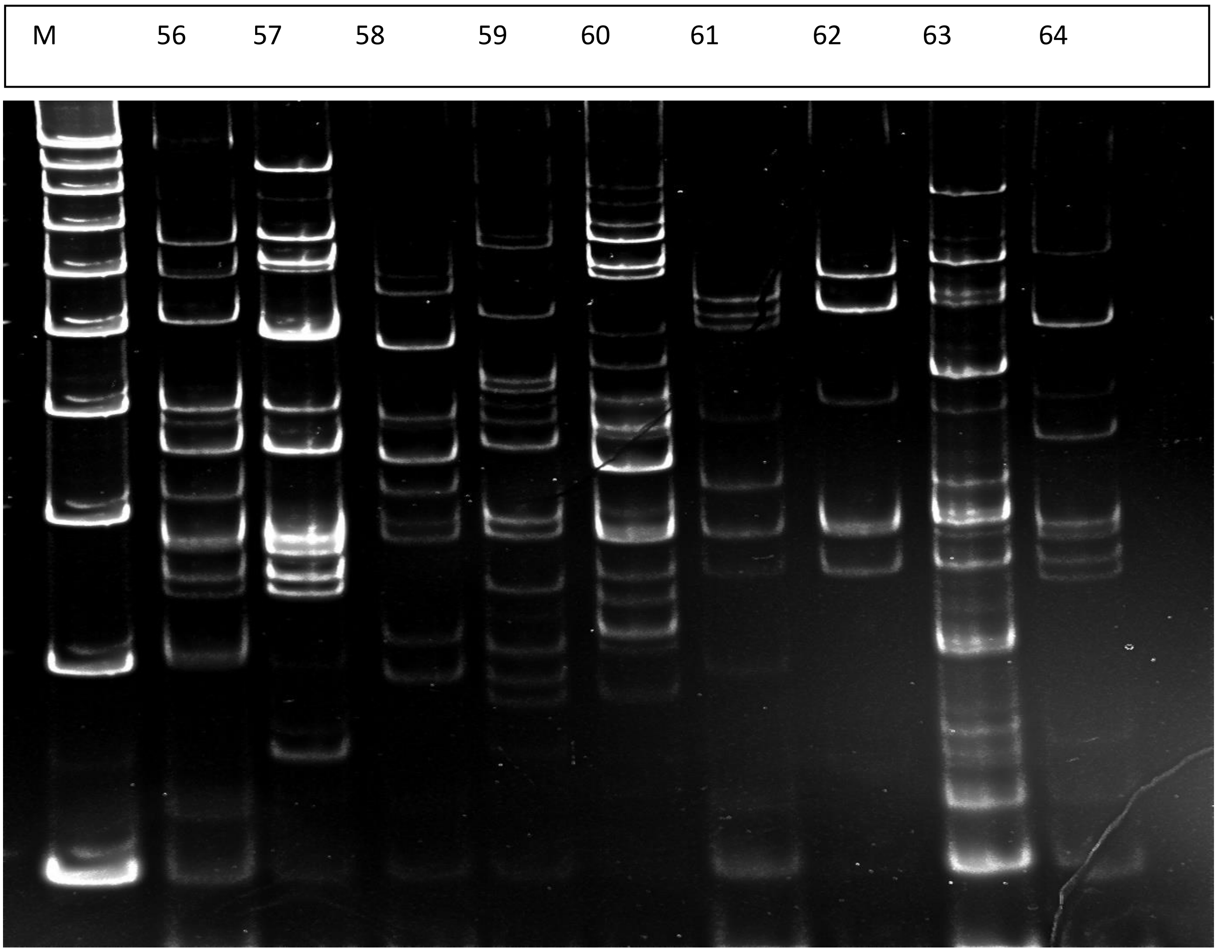
M
56
57
58
59
60
61
62
63
64

M 1 2 3 4 5 6 7 8 9 10 11 12 13 14 15 16 17 18 **M** 192021222324252627282930313233343536; **M** 37 38 39 40 41 42 43 44 45 46 47 48 49 50 51 52 53 54 55; **M** 56 57 58 59 60 61 62 63 64

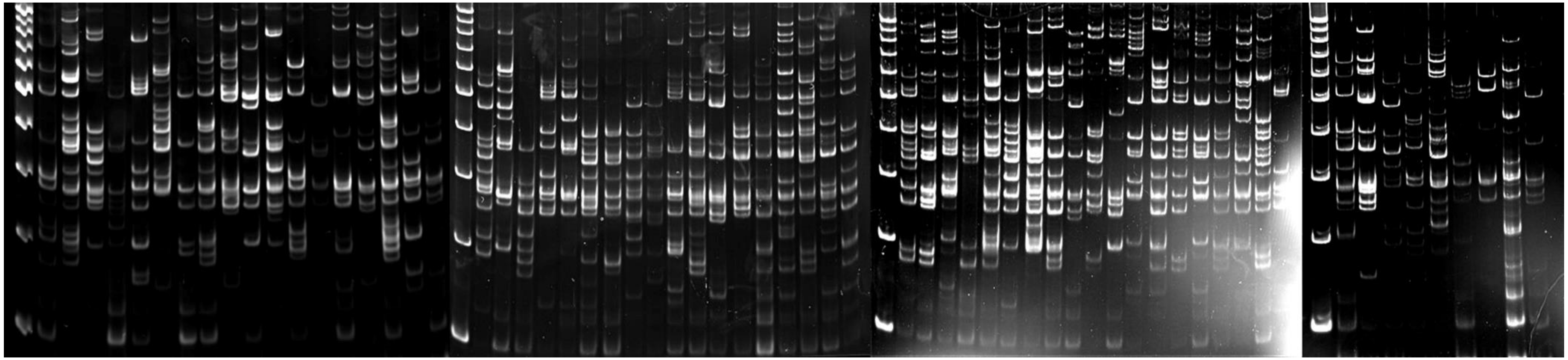

S4_raw_images: Genotyping of isolates by PCR fingerprinting to establish genetic relationships between *E. coli* strains (1-64). Isolates from blood of patients with urosepsis. M-DNA size marker: 100-1000. Electrophoretic separations were performed on 6% polyacrylamide gels against 1 x TBE buffer.
